# The Neural Analysis Toolkit Unifies Semi-Analytical Techniques to Simplify, Understand, and Simulate Dendrites

**DOI:** 10.1007/s12021-025-09766-x

**Published:** 2026-04-14

**Authors:** Willem A. M. Wybo

**Affiliations:** Peter Grünberg Institute 15 – Neuromorphic Software Ecosystems (PGI-15), Jülich Research Center, Dennewartstraße 25-27, 52068 Aachen, Germany

**Keywords:** Dendritic simplification, Dendritic computation, Biophysical neuron models, Cable theory

## Abstract

While simulating compartmental dynamics in response to various input patterns is the prevalent technique for understanding dendritic computation, a great deal can be learned from classical analytical methods that provide solutions for the dendritic voltage. For example, such solutions are needed to simplify spatially extended neuron models, to understand frequency-dependent response properties, to elucidate the interaction between synaptic inputs, and hence to reveal the effective compartmentalization of dendrites into functional subunits. Nevertheless, these methods have not been implemented in modern software tools. This work describes the NEural Analysis Toolkit (NEAT), a Python toolbox that implements classical algorithms to compute response properties of spatially extended neuron models, and that leverages these algorithms to simplify them. Packaged with this are a range of useful utilities to plot morphologies and spatial quantities defined on the morphology, to distribute locations on the morphology, and to select parts of the morphology to e.g. apply morphological ablations or alter the membrane properties. The resulting models can be exported to NEURON and NEST, two commonly used simulators, the former focused on detailed single neuron model simulations, and the latter geared towards distributed network simulations. As a consequence, this toolbox provides a missing link between single neuron computation and large-scale network analysis, substantially facilitating the study of the role of dendritic computation in shaping emergent network dynamics.

**Supplementary Information:**

The online version contains supplementary material available at 10.1007/s12021-025-09766-x.

## Introduction

Computational neuroscience is driven by software tools. To simulate networks of neurons, and understand the effects of synaptic plasticity, computational neuroscientists typically rely on NEURON to investigate morphologically detailed neurons (Carnevale and Hines, 2004), on NEST when the efficiency of network simulations is of essence (Gewaltig and Diesmann, 2007), and on Brian when model customization and ease of implementation is of primary importance (Stimberg et al., 2019). Over the years, a rich ecosystem of models has been developed for each of these tools. In NEURON, the primary use case is spatially extended neuron models, where the membrane is equipped with currents whose conductance depends on the local membrane potential and/or presynaptic transmitter release, and whose dynamics can be made arbitrarily complex through the .mod model description language (see e.g. ModelDB Hines et al., 2004). In NEST, the collection of available neuron models has so far been more simple, with a range of predefined neuron models provided by default, and the recent development of a model-description language which, in its implicit assumptions, was geared towards point neurons (Blundell et al., 2018). However, the algorithms that transmit spikes between neurons have been honed to enable a highly efficient communication on massively parallel computing systems (Kunkel et al., 2014; Jordan et al., 2018). Finally, Brian offers much freedom in the model definition, converting arbitrary differential equations in C++ code, but does not offer the advanced parallellisation functionalities to simulate large networks (Stimberg et al., 2019). These simulation tools, therefore, encourage development of specific branches of computational neuroscience, by each creating a niche of applications in which model development is easy, while research that falls outside of these areas of application is typically hard, and requires development of extensive problem-specific research codes.

One of the areas of research that falls outside of the realm of applicability of standard software tools, but that is fundamental towards understanding brain function, is that of understanding the effective degree of dendritic complexity at which brain circuits operate (Branco and Häusser, 2010; Major et al., 2013; Poirazi and Papoutsi, 2020). While simulators like NEURON (Hines, 1984; Carnevale and Hines, 2004) and Arbor (Abi Akar et al., 2019; Akar et al., 2023) implement the discretisation of neural morphologies into coupled ordinary differential equations through the 2nd order finite difference approximation, internally effectively constructing a representation of the neuron model as a series of coupled electrical compartments, they offer no native tools to substantially simplify these models. While many simplified, rate-based neuron models have been proposed that convert the dendrite into multi-layer perceptron-like models (Poirazi et al., 2003; Jadi et al., 2014; Behabadi and Mel, 2014; Ujfalussy et al., 2018) – leading authors to explore such neuron models in a machine learning context (Jones and Kording, 2021; Beniaguev et al., 2021; Chavlis and Poirazi, 2024) – this work focuses on descriptions in terms of electrical compartments. Such description result in spiking models, and offer a biophysical interpretation for their parameters. In a top-down approach, reduced compartmental models are often constructed as combinations of few coupled compartments, whose conductance parameters are optimized to reproduce chosen dendritic computations (Naud et al., 2014; Quaresima et al., 2022; Pagkalos et al., 2023; Zhang et al., 2024; Pastorelli et al., 2025; Naghieh et al., 2025). On the other end of the spectrum, bottom-up approaches start from highly detailed models, simplifying them by requiring that the reduced model conserves geometrical quantities like surface area (Hendrickson et al., 2011; Marasco et al., 2013), or electrical properties like attenuation (Destexhe, 2001) or transfer impedance (Amsalem et al., 2020). Such approaches can essentially be thought of as finding appropriate rescaled conductances for the compartmental currents. Prior work has shown that the inverse of the dendritic resistance matrix, evaluated at dendritic sites of interest, yields precisely the desired conductance parameters (Wybo et al., 2021). The resulting simplification methodology is useful for both the top-down approach (Pastorelli et al., 2025) as well as the bottom-up approach (Wybo et al., 2021).

The simplification algorithm by Wybo et al. (2021) requires evaluating large numbers of such resistance matrices around different expansion points, to generalize well to non-linear dynamical regimes. Such analytically computable quantities furthermore yield insight into response properties of the neuron. Input and transfer resistance matrices yield insight into the compartmentalisation of the neuron model into functional subunits (Cuntz et al., 2010; Wybo et al., 2019), and in spatial summation across the dendritic tree (Li et al., 2019). Input and transfer impedances yield insight in the neuron’s spatio-temporal responses to different input frequencies (Laudanski et al., 2014). Computing the time-scales of the neural response kernels provides information on the effect of dendritic morphology on input integration and action potential firing (Eyal et al., 2014), while computing conductance loads explains spike feature variability (Hay et al., 2013) and excitation-inhibition interactions (Gidon and Segev, 2012). Standard software tools, however, do not provide analytical algorithms to compute such response properties. While these quantities can be derived approximately from simulation results, they can be computed with more efficiency and/or accuracy through analytical algorithms, such as Koch’s (Koch and Poggio, 1985) or Abbotts (Abbott et al., 1991; Coombes et al., 2007; Caudron et al., 2012) algorithm to compute input and transfer resistances and impedances (Koch and Poggio, 1985), or Major’s algorithm to compute the separation of variables expansion (Major et al., 1993, 1993b; Major, 1993; Major and Evans, 1994), which provides response timescales and their associated spatial profiles (Holmes et al., 1992).

Supported by the NEST initiative, this paper presents the NEural Analysis Toolkit (NEST-NEAT), which as it primary application, aims to make neuron simplification easy. In doing so, NEST-NEAT thus provides a bridge between single- and multicompartment modeling. In general, there is a need to make interacting with complex dendrite models easier (Makarov et al., 2024). NEST-NEAT relies largely on Python, and provides a Pythonic and convenient of way of defining morphologically extended neuron models, reading the commonplace ‘.swc’ format for specifying morphologies into an internal representation of cylindrical sections. NEST-NEAT also provides a number of general routines interacting with the morphology, distributing and defining sets of locations, and for specifying biophysical mechanisms. Furthermore, NEST-NEAT implements Koch’s algorithms to compute input and transfer impedances in the Fourier domain (Koch, 1984), and appropriate custom inverse transform algorithms to convert these frequency-domain impedances to response kernels in the time domain. NEST-NEAT also implements Major’s separation of variables algorithm (Major et al., 1993, 1993b; Major, 1993; Major and Evans, 1994), to compute the response timescales of neuron models and their associated spatial profiles. NEST-NEAT uses these analytical algorithms to implement Wybo’s simplification algorithm (Wybo et al., 2021), which can be used to derive accurate simplifications of arbitrary complexity. Further, this paper describes novel additions to the toolbox, which include the ability to incorporate concentration dynamics in the model analysis and simplification pipeline, as well as to the ability export full and reduced models to NEST – next to the already implemented NEURON export – for simulation.

## Design and Implementation

### An Overview of NEAT

**Fig. 1:**
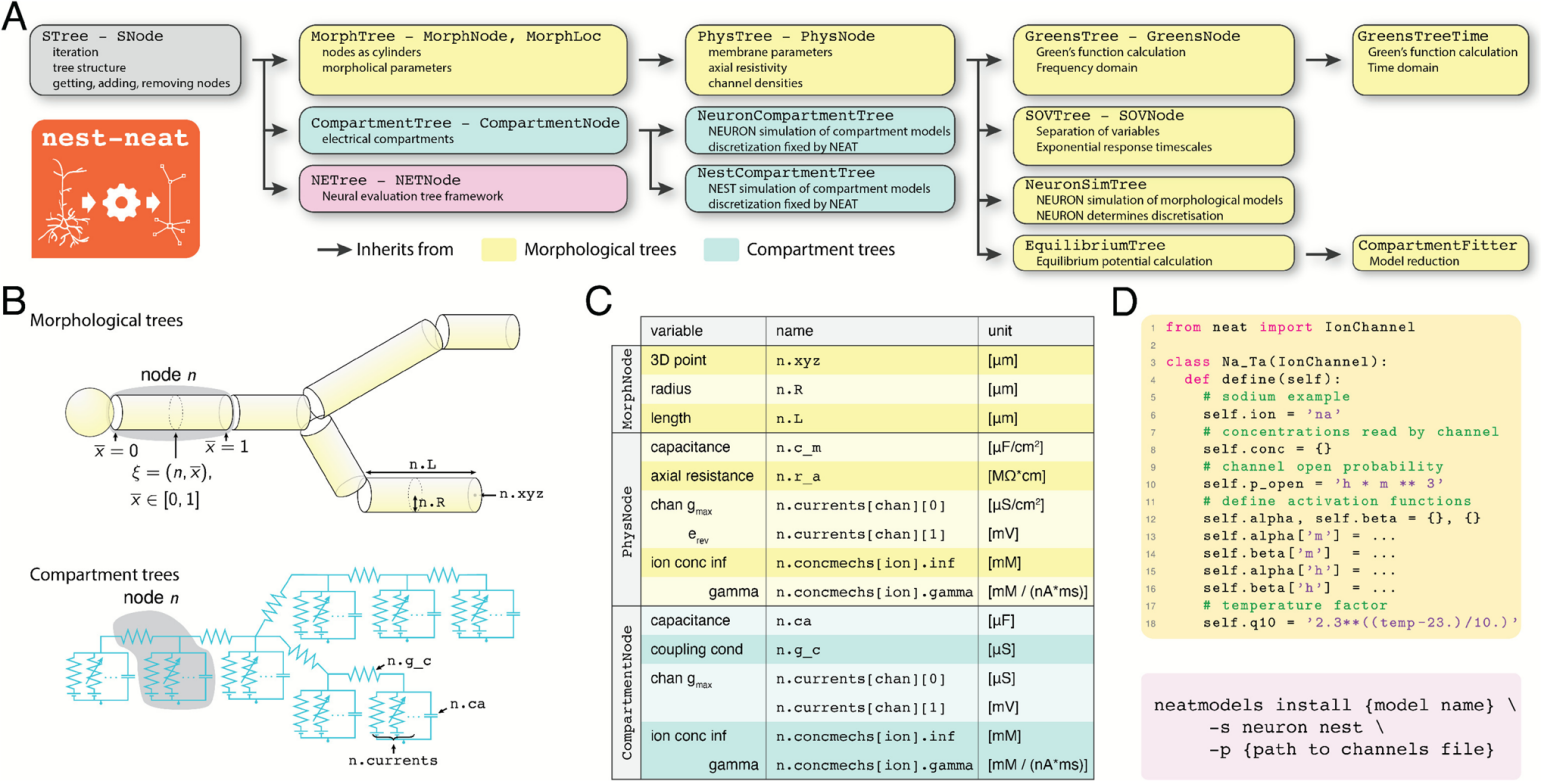
The structure of NEAT. **A:** Overview of the inheritance structure of NEAT’s trees and associated node classes. In morphological trees (yellow), nodes represent cylindrical sections, whereas in compartment trees (blue), nodes represent electrical compartments. The neural evaluation tree (NETree) is a specialty tree developed to analyze independence of subunits (Wybo et al., 2019). **B:** In morphological trees (top), the node index n refers to a cylindrical section with a length n.L, radius n.R and the xyz-coordinate of the endpoint n.xyz. A further coordinate $\overline{x} \in [0,1]$ is needed to fully specify a location. In compartment trees (bottom), only a node index n is required. A compartment node n is coupled to its parent through the coupling conductance n.g_c. **C:** Table of critical parameters stored at each node, how to access them in the data structures, and their units. Note that nodes have the same inheritance structure as trees, so that a PhysNode inherits from MorphNode, and therefore also contains all its parameters. **D:** Code example of an IonChannel in NEAT. At present, NEAT only supports Hodgkin-Huxley type channels in its analytical calculations, from which ion channel simulation code for NEURON and NEST can be generated. **E:** To generate simulation code for NEURON and/or NEST, NEAT provides the neatmodels terminal script, which compiles groups of ion channels either into *.mod-files or into *.nestml-files

As the dendritic arborizations of neurons can be represented as tree graphs, NEST-NEAT (referred to simply as NEAT in the remainder of this text for brevity) is built almost entirely on this data structures (Fig. 1A). A tree-graph is a graph without loops. As such, one node can be understood to be the root of the tree. Each node then stores a number of child nodes, and a reference to its parent (None in case of the root). NEAT features three types of tree classes: abstract, compartmental and morphological. The abstract tree class STree is the base class from which all trees inherit, and implements basic functionality to iterate through trees, to add and remove nodes, and queries to return specific collections of nodes (e.g. the subtree of a specific node). In the compartment tree classes (CompartmentTree and derived classes), each node is assumed to be a single electrical compartment coupled to its parent through a coupling conductance (Fig. 1B). Finally, in the morphological tree classes (MorphTree and derived classes) each node is assumed to signify a cylindrical section of neurite, which connects the xyz-coordinate of the parent node to its own xyz-coordinate.

In NEAT, the various tree classes either add a layer of complexity, or implement a different functionality. For instance, NEAT’s MorphTree – initialized from the de-facto standard .swc-format (Ascoli et al., 2007)– stores the morphology of the neuron, and provides functionality to interact with it, such as by distributing locations on the morphology. However, it does not store physiological parameters; this is implemented by NEAT’s PhysTree. The PhysTree inherits from MorphTree, and adds functionality to set physiological parameters, such as passive parameters (axial resistance, membrane capacitance, leak conductance and reversal) or ion channel densities and concentration mechanics. A PhysTree thus represents a detailed biophysical neuron model. A number of classes then inherit from PhysTree to implement specific computations, such as the SOVTree, which computes the separation of variables expansion through Major’s algorithm, the GreensTree, which computes the Green’s function in the Frequency domain through Koch’s algorithm, the GreensTreeTime, which transforms frequency domain results to the time domain, the EquilibriumTree, which computes the equilibrium potential throughout the tree, and finally the CompartmentFitter, which derives simplified neuron models and returns them as CompartmentTree instances. As all these trees inherit from PhysTree, they can be instantiated using the same routines to specify morphology and physiology. However, if a tree is first built as one specific tree class, it would be cumbersome to construct it in the same way if another computation is needed. To that purpose, NEAT implements a copy-construct functionality, so that any tree type can also be instantiated from any other tree class instance.

Finally, to simulate neuron models, NEAT implements the NeuronSimTree, which inherits from PhysTree and exports full morphological models (where nodes are combinations of cylinders) to NEURON. NEAT also defines the NeuronCompartmentTree and NestCompartmentTree, which inherit from CompartmentTree and export reduced neuron models to NEURON resp. NEST.

Morphological reconstructions, specified in the .swc format, often feature unbranched sections of dendrite, consisting of many nodes with identical radii, while the physiological parameters of the model are also identical for these nodes. In principle, such a section could be replaced by a single cylinder, without changing the underlying mathematical description. However, NEAT’s MorphTree aims to preserve a tree structure that is a one-to-one mapping to the underlying .swc datafile, i.e. every node corresponds to an underlying .swc node, and represents a cylinder with as radius the radius provided in the .swc file, connecting the .swc xyz-coordinate to its parent’s xyz-coordinate. To conserve this relationship, but also improve computational efficiency, morphological trees in NEAT have the set_comp_tree() function. This function internally constructs a coarse-grained tree, referred to as the computational tree, where nodes with identical radii and physiological parameters (up to a user-specified tolerance) are grouped in a single cylindrical section. This coarse grained tree is activated with the as_computational_tree() context manager. Compute-intensive functionalities activate this computational tree automatically. Note that morphologies can also be resampled arbitrarily, through the create_new_tree()function.

### NEAT’s Ion Channels

NEAT provides a simple and lightweight ion channel description format. This format admits ion channels of the Hodgkin-Huxley (HH) type (Hodgkin and Huxley, 1952), by which we mean that the channel currents are of the form

1 $$\begin{aligned} i_{c} = \overline{g}_{c} \, o_c(\textbf{y}_c(t)) \, (v(t) - e_c), \end{aligned}$$

where $\overline{g}_{c}$ the channel’s maximal conductance density, $e_c$ the reversal potential, and $o_c$ the opening probability as a function of the channel’s state variables $\textbf{y}_c(t)$. The state variables then evolve according to first-order ODEs that are non-linearly coupled to the local membrane voltage *v*(*t*), which can be specified in two ways: either

2 $$\begin{aligned} \dot{y}_{ci}(t)&= \alpha _{ci}(v(t)) \, (1 - y_{ci}(t)) + \beta _{ci}(v(t)) \, y_{ci}(t), \hspace{4mm} \text {or} \end{aligned}$$

3 $$\begin{aligned} \dot{y}_{ci}(t)&= \frac{y_{ci,\infty }(v(t)) - y_{ci}(t)}{\tau _{y_{ci}}(v(t))}. \end{aligned}$$

Note that the functions $\alpha _{ci}(\cdot ), \beta _{ci}(\cdot )$ or $y_{ci,\infty }(\cdot ), \tau _{y_{ci}}(\cdot )$ may additionally depend in a user-defined fashion on ion concentrations. Note furthermore that the open probability can be any expression featuring the channel’s state variables.

Implementation-wise, one defines channels as subclasses of IonChannel, NEAT’s ion channel base class (Fig. 1D, top). Then, it suffices to write a define() function, which defines the open probability p_open as a channel attribute that is a SymPy expression or a Sympy-readable string (Meurer et al., 2017). Similarly, the functions $\alpha _{ci}(v), \beta _{ci}(v)$ or $y_{ci,\infty }(v), \tau _{y_{ci}}(v)$ governing state variable evolution are defined as dictionaries with as keys the state variable names and as values the SymPy expressions or SymPy-readable strings. Note that the dictionary keys have to correspond to state variable names featured in the open probability p_open. A temperature dependence factor q10 for the reaction rates can be specified separately, as well as a custom reversal e (if not provided, a NEAT default is used based on the ion the channel is permeable to (Na: 50 mV, K: -85.0 mV, and Ca: 50.0 mV). State variable evolution can also depend on a set of ion concentrations, which need to be specified in the conc attribute.

NEAT’s IonChannel class features functions that provide the necessary quantities to NEAT’s analytical algorithms, as well as code generators that write .mod-files for NEURON and .nestml files for NEST, ensuring that both the analytical computations and the models in various simulators use the exact same underlying channel dynamics. Moreover, groups of NEAT’s ion channels can easily be compiled into ‘models’ for NEURON or NEST, using the ‘neatmodels’ script (Fig. 1D, bottom), which then allows identical models to be simulated in both simulators.

### The Coordinate System of NEAT

To fully specify a location on a branching morphology consisting of cylindrical sections, we need both a node index *n*, and a spatial coordinate $x \in [0, L_n]$, which runs from 0 at the proximal end to $L_n$ – the node’s length – at the distal end. For instance, we may specify the membrane voltage at node *n*, position *x* as $v_n(x,t)$. As the lengths of nodes may vary depending on the morphology specified in the .swc source file, it is not convenient to refer the actual *x*-coordinate in practice. NEAT therefore uses a normalized spatial coordinate $\overline{x} \equiv \frac{x}{L_n} \in [0,1]$. Thus, a location $\xi$ in NEAT is always specified as a tuple of node index and normalized x-coordinate, i.e. $\xi = (n, \overline{x})$. To avoid cluttered subscripts in the mathematical notations, we will adopt the convention that if a quantity, e.g. membrane voltage, features a full location coordinate $\xi = (n, \frac{x}{L_n})$, the node index will not be made explicit, i.e. $v(\xi ,t) \equiv v_n(x,t)$.

Locations in NEAT are implemented with NEAT’s MorphLoc class, and are always defined with respect to a reference tree (a MorphTree or derived class). These locations obey the continuity constrained at the connection points between cylinders: if node *n* is the parent node of *m*, then $(n, x=1) \equiv (m, x=0)$. Furthermore, from the user perspective, these location objects are invariant to the coordinate transformation induced by the switching to the computational tree-context; internally, the MorphLoc constructs and stores a coordinate for the computational tree that corresponds to the same location on the original tree.

Although NEAT’s location scheme with nodes resp. x-coordinates bears resemblance to how NEURON’s sections resp. segments are accessed, the segment coordinate in NEURON will gives access to the nearest segment on the section. Thus, the continuous [0, 1] values essentially access discrete segments. In NEAT, the x-coordinate is truly continuous, and e.g. analytical solutions for the cable equation are computed exactly at the associated location.

### Model Construction

**Fig. 2:**
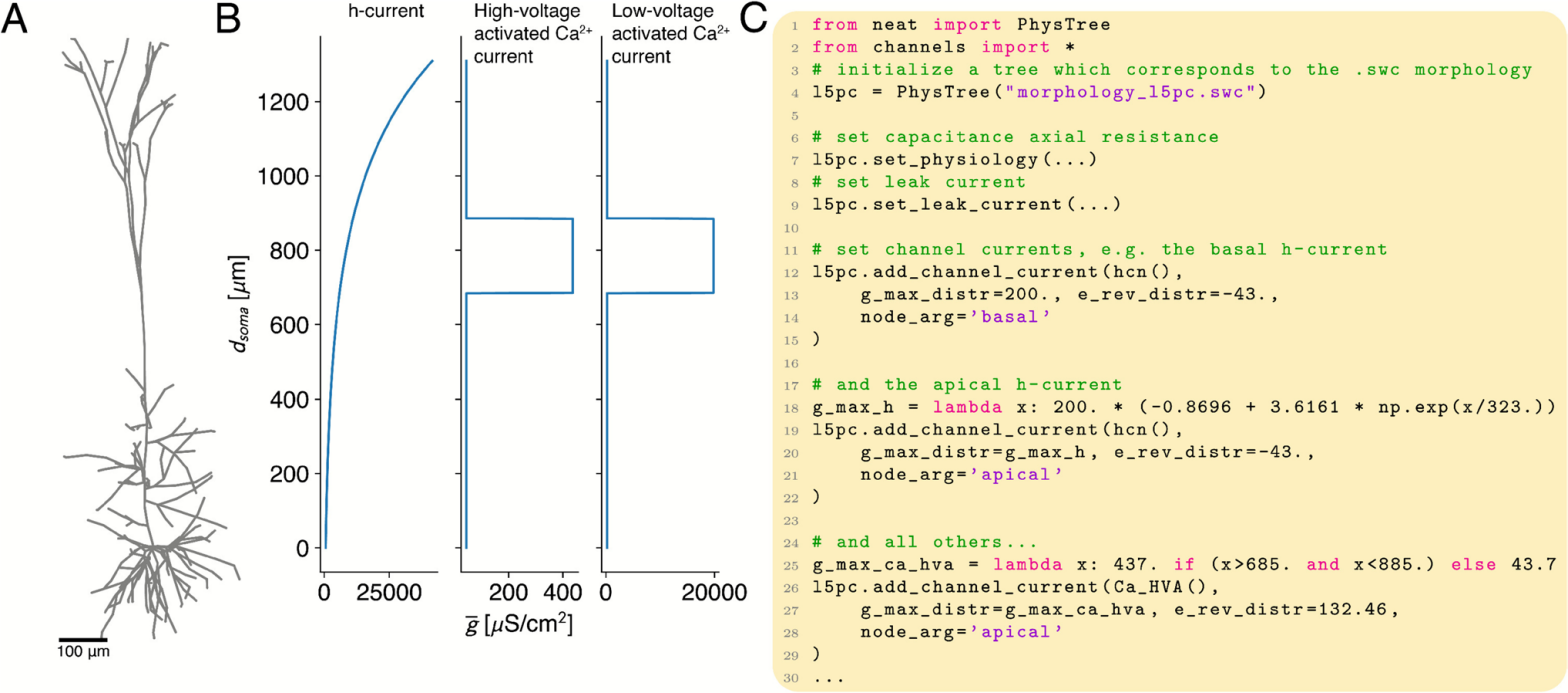
Constructing morphological models in NEAT. **A:** NEAT loads morphologies as .swc files. **B:** Ion channel densities are specified as a function of the distance from the soma (in $\mu$m). **C:** Code example of how to load a morphology and how to distribute ion channels. Parameters such as conductance density (g_max_distr) and reversal potential (e_rev_distr) can be specified as floats, callables where the argument signifies the distance to the soma, or on a per node basis as dictionaries with node indices as keys. Note that the ion channels have to be provided as neat.IonChannel objects (here imported from channels.py)

All operations in NEAT revolve around a tree graph structure that stores the morphological and electrical parameters of the neuron model. If the analysis to be performed is limited to the morphological structure of the neuron, a MorphTree instance suffices. However, as soon as the electrical behaviour of a cell needs to be studied, physiological parameters need to be added to the neuron model. NEAT implements an intuitive system to build models rapidly. To illustrate the functionalities of NEAT, we have implemented a classical layer 5 pyramidal cell (L5PC) model by Hay et al. (2011) (Fig. 2A).

First, any morphological tree class instance can be constructed from an ‘.swc’ file, the de-facto standard for describing neuron morphologies (Fig. 2A,C, Ascoli et al. (2007)). To enable usage of the analytical solutions of the cable equation, it should be noted that NEAT interpretes the ‘.swc’ nodes as cylindrical sections of neurites, a slight departure from their original intended interpretation as truncated cones. Constructing the model in this way results in a tree consisting of nodes whose indices correspond to the node indices in the ‘.swc’ datafile (Fig. 1C), and whose morphological parameters – cylinder radius, cylinder length, and spatial coordinate of the most distal point (Fig. 1C) – contain the values from the underlying datafile.

At this point, the NEAT model only contains morphology information. To turn this into a full, biophysical neuron model, physiological parameters such as membrane capacitance, axial resistance, leak conductance densities and reversals, and ion channel conductance densities and reversals (Fig. 1C) have to be set. NEAT’s PhysTree and derived tree classes provide convenient functions for these tasks, that provide a great deal of flexibility. They allow these parameters to be set uniformly by passing a single float, as a function of the distance to the soma by passing a callable function, or on a per-node basis by passing a dictionary with node indices as keys (Fig. 2B,C). Further, these functions can be applied to specific groups of nodes using the node_arg argument.

As many algorithms in NEAT perform analytical calculations on cylinders, ensuring that the number of cylinders in a neuron model is minimal is beneficial for computational efficiency. Often, situations occur where a linear section of dendrite consists of many ‘.swc’ nodes, each with the same radius and physiological parameters. These small cylindrical segments can be grouped into a single cylinder encompassing the whole dendritic segment. However, converting a tree object in this way would distort the morphology, and also result in a mismatch with the underlying ‘.swc’ data file. For these reasons, morphological trees in NEAT contain a coarse-grained computational tree under the hood. This tree is computed by calling the set_comp_tree() function, which assesses the differences in parameters values between cylinders to determine whether they can be united in a single, coarse-grained cylinder. For this reason, the function should only be called after all parameters have been set, i.e *after model construction is complete*. NEAT then provides the as_computational_tree() context manager, which activates the computational tree. Note that the associated coordinate transformations are all performed internally by NEAT, so that the user facing interface always expects coordinates that reference the original tree. Note furthermore that modifying *any* physiological or morphological parameters of the neuron model, will automatically result in the deletion of the computational tree, as it can not be guaranteed to be the same after the modification. Note finally, that many of NEAT’s analytical computations will automatically attempt to activate the computational tree context, and raise an error when it has not been set.

### Interacting with NEAT Trees

**Fig. 3:**
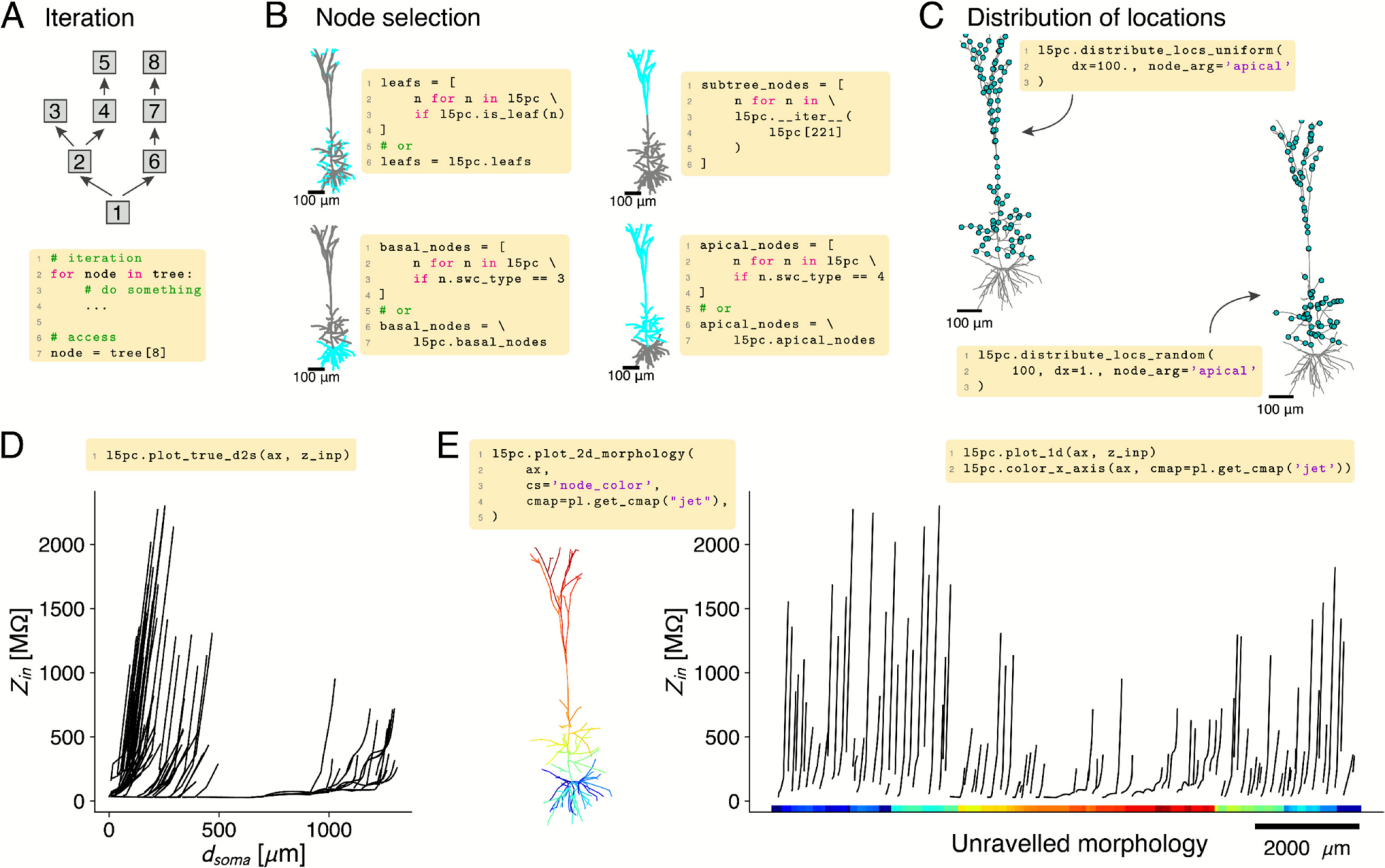
Interacting with morphological models in NEAT to access individual nodes and groups of nodes, to distribute locations, and to plot location-dependent quantities on the morphology. **A:** NEAT trees provide a depth-first iterator, which proceeds to the end of a branch first, and then continues at the nearest bifurcation where there are child nodes that have not yet been traversed (top). Individual nodes can be accessed by their index (bottom). **B**: NEAT allows the flexible selection of groups of nodes through its iterator. Standard groups of nodes, such as the distal tips (leaf nodes, top left), basal nodes (bottom left), or apical nodes (bottom right), can be accessed through convenient attributes. Providing a node to the iterator (top right) starts the iteration from that node, providing access to a subtree of the dendrite. **C:** NEAT’s MorphTree and derived classes provide a number of functions to distribute locations, which could represent synaptic input sites or compartment sites, on the tree. These functions provide for instance evenly spaced locations (top left) or randomly distributed locations (bottom right). **D:** Functionality to plot spatial quantities as a function of the distance to the soma, here illustrated with the input resistance of the L5PC model. **E:** Unraveling of the morphology to plot quantities (here illustrated with input resistance as in D). A matching color code between morphological locations (left) and x-axis (right) clarifies the location of each branch

NEAT provides a Pythonic way of interacting with its trees. Individual nodes can be accessed by their swc-index (Fig. 3A), and NEAT also provides a depth-first iterator for accessing all nodes (Fig. 3A). This allows for the selection of specific groups of nodes according to user-defined criteria (Fig. 3B).

As described previously, locations on the morphology, which could for instance represent synapse sites or compartment sites, are encoded as a node index and normalized x-coordinate. NEAT provides the MorphLoc class for storing such locations, which also stores a reference to the tree on which the location is defined. As such, MorphLoc instances are context-aware, and return either original tree coordinates or computational tree coordinates, depending on which tree is active. Nevertheless, all functions that require locations as input also accept simple tuples (node_index, x) or dictionaries {’node’: node_index, ’x’, x}, and interpret them in the coordinate system of the original tree. They are then converted internally to MorphLoc instances, so that users are not obliged to instantiate them explicitly. NEAT further provides functionality to distribute locations on the morphology, for instance in a uniformly spaced or random fashion (Fig. 3C). These functions return lists of locations, i.e. containing MorphLoc instances.

As one of NEAT’s purposes is to analyse dynamics in dendrites, NEAT allows visualization of quantities that depend on the location, such as input or transfer resistances, membrane voltage, concentrations, etc. For instance, such quantities can be visualized as a function of the distance to the soma (Fig. 3D). While this provides an accurate view along each branch, it can result in a plot that is hard to parse for complex morphologies that branch frequently. For this reason, NEAT also allows unravelling the morphology on a one-dimensional axis (Fig. 3E). In this view, x-positions can be matched to the location on the neuron through the color code of the x-axis. Finally, straightforward recipes to color code quantities that depend on dendritic location directly on the neuron are outlined in the documentation.

## Results

### Response Kernel Calculation

The response kernels, input and transfer impedances, and input and transfer resistances of a neuron model reveal much about how the dendritic tree computes. These are all ways of expressing the Green’s function of the linearised neuron model (see supplement). In particular, response kernels have provided insight into compartmentalisation of dendrites into computational subunits (Wybo et al., 2019), and into the temporal filtering characteristics of dendritic trees (Eyal et al., 2014; Holmes et al., 1992). Input and transfer impedances yield insight into frequency-dependent response properties, such as preferred frequencies along the dendritic arborisation (Laudanski et al., 2014), and the frequency dependent electrical size of the dendritic tree (Koch, 1998; Zador et al., 1995). Finally, input and transfer resistance have shown to be a strong indicator of compartmentalisation (Cuntz et al., 2010; Gidon and Segev, 2012; Wybo et al., 2019), yielding information on spatial interactions between conductances on dendritic trees and the integrative properties of the dendrite. In NEAT, they are furthermore used extensively in the simplification algorithm. Thus, analytical algorithms to compute the Green’s function are the foundation of NEAT.

**Fig. 4:**
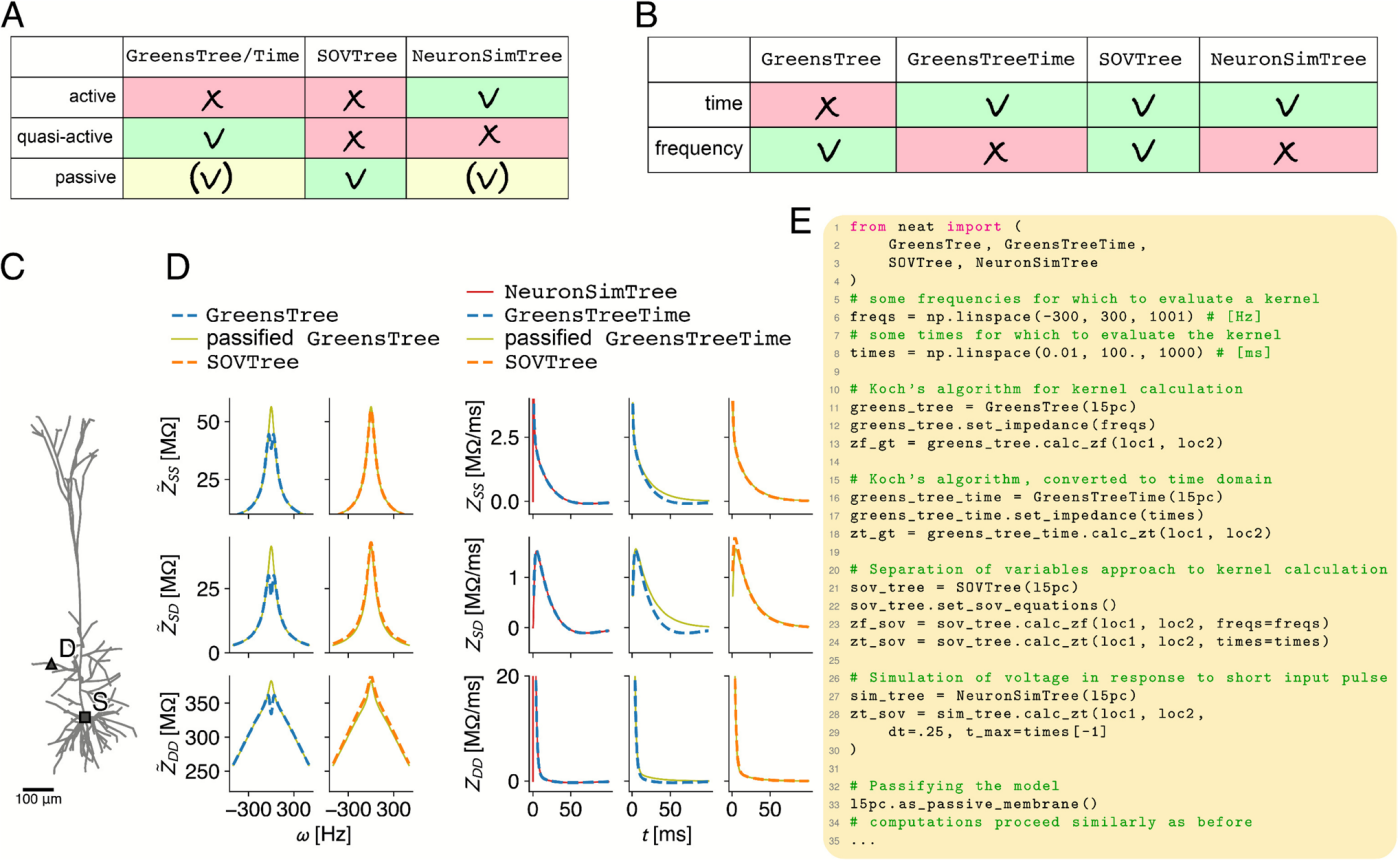
Overview of different input and transfer response kernel calculation methods implemented in NEAT. **A:** Implicit assumption made by each tree class, i.e. the approximation the respective analytical algorithm makes about the membrane dynamics (green). GreensTree/Time and NeuronSimTree can also use the passive assumption, but have to be converted explicitly using the as_passive_membrane() function (yellow). **B:** Overview of whether the kernel calculation method implemented by the tree class computes the response kernels in the time and/or frequency domain. **C:** Exemplar L5 pyramidal cell morphology with a dendritic site (D) and the soma (S). **D:** Input response kernel at the soma (top), transfer response kernel between soma and dendrite (middle), and input response kernel in the dendrite (bottom), computed at site S and D in C. Left, response kernels in the frequency domain. Note that the GreensTree by default uses the quasi-active approximation, whereas the SOVTree can only use the passive approximation (cf. A). The GreensTree can be passified, however, after which the kernels coincide with those computed by the SOVTree. Right, response kernels in the time domain. Here, kernels can also be computed by an explicit simulation of the neuron model, by measuring the voltage response following a small and short input current pulse. These kernels coincide with the quasi-active ones computed by the GreensTreeTime. **E:** Code example of how to compute the different kernels. With GreensTree/Time, all internal impedances (essentially, the boundary conditions for all cylindrical segments, cf. supplement) need to be computed first using the set_impedance() function. To compute the kernels as superpositions of exponentials, the transcendental equation needs to be solved first, and then the equations to compute the associated spatial modes. Both steps are performed in the set_sov_equations() call (cf. supplement)

Each algorithm requires different intermediate quantities to be stored on the tree graph, and therefore is implemented by a separate tree class. NEAT provides three ways of computing the Green’s function: *(i)* using Koch’s algorithm, which assumes the quasi-active approximation (with GreensTree / GreensTreeTime), *(ii)* using Major’s algorithm to find the separation of variables solution, which only works for passified membranes (see supplement, with SOVTree), and *(iii)* through simulations of the full neuron model (with NeuronSimTree), where the voltage in response the small, short current pulses is measured (Fig. 4A). Koch’s algorithm operates in the frequency domain, but GreensTreeTime implements NEAT’s algorithm to convert these impedances to time domain kernels. As Major’s algorithms yields superpositions of exponentials, it applies both to the frequency and time domains. Finally, simulations only yield time domain results (Fig. 4B).

To illustrate the different algorithms, we compute the response kernels at a dendritic site, the soma, and between soma and dendrite on the L5PC (Fig. 4C), which features a total of seven active ion channels on the apical tree. It can be noted in this regard that impedances computed from the passified tree (i.e. with the explicitly passified GreensTree or with the SOVTree) are always low-pass filters, and impedances from the quasi-active tree can be band-pass filters (Fig. 4D, left). Furthermore, the passified and normal kernels coincide in the high-frequency regime; on short time-scales (below 1 to 10 ms), the dendritic voltage response is dominated by the axial currents, which are captured equally well in the passified model as in the quasi-active one. For this reason, the passified model is an excellent predictor of the compartmentalisation of dendritic trees into computational subunits (Wybo et al., 2019). In the low-frequency regime - and therefore on longer time-scales ($> 10$ ms) - the response kernels from quasi-active and passive models differ substantially, the former reflecting a preferred frequency characteristic of the dendritic h-current (Remme and Rinzel, 2011; Rich et al., 2021). In the time-domain, the quasi active response kernels from Koch’s algorithm (GreensTreeTime) agree well with those computed through simulations (NeuronSimTree, Fig. 4D, right).

So which algorithm should one choose? All algorithms can be called in few lines of code (Fig. 4E). For accuracy and computational efficiency, Koch’s algorithm is the preferred choice. The separation of variables method yields a nice and useful representation as a superposition of exponentials, but is computationally less efficient. Large neurons with many branches furthermore produce many equalising timescales (zeros of the transcendental function) in a similar range, some of which can be missed by the zero finding algorithm. This results in a deterioration of accuracy. The simulation based approach is accurate, but computationally inefficient. It also can not easily be applied to other expansion points than the resting state, whereas in Koch’s algorithm the linearizations can be computed around any arbitrary combination of voltage and state variables. It should be noted that NEURON also provides an impedance analysis tool, which computes the Fourier transform of its internal compartmental description of the NEURON model and inverts the associated matrix using a Jacobi iteration. By consequence, this impedance analyzer is discrete in space. NEAT’s algorithms are continuous in space and thus allow the study of impedances at a sub-compartmental level.

### Model Reduction

**Fig. 5:**
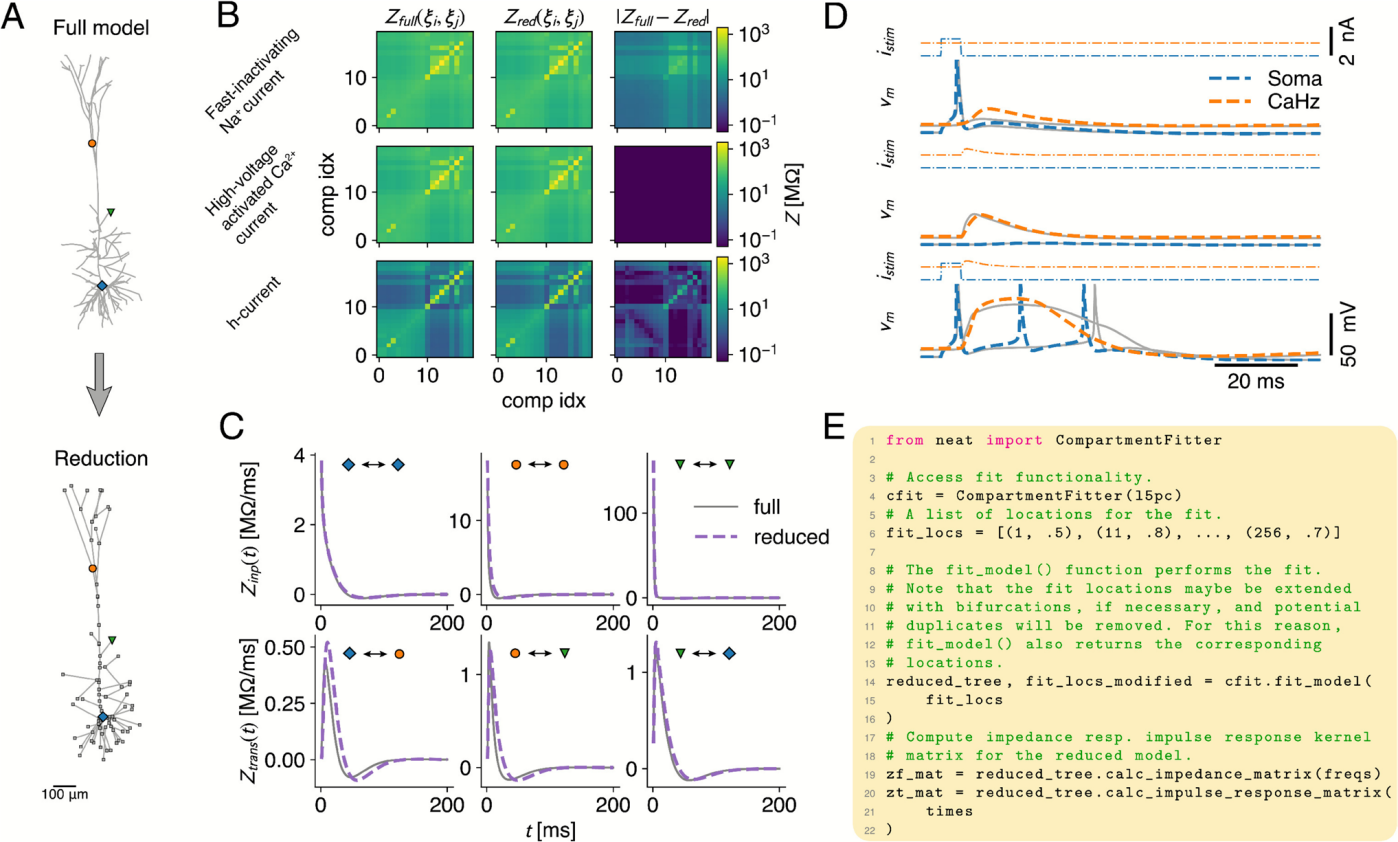
Reducing morphological neuron models with NEAT. **A:** Morphological models – consisting of cylindrical sections – can be converted to simplified compartment models, where the compartments represent user-defined locations on the morphology. **B:** Reductions with NEAT fit the conductance parameters in each compartment to reproduce resistance matrices, i.e. the matrix *Z* where $Z_{ij} = \tilde{Z}(\xi _i, \xi _j, \omega =0)$. For each ion channel separately, multiple resistance matrices are compute at different activation levels to ensure generalization to the full non-linear dynamics. **C:** Reductions with NEAT fit the capacitance parameter in each compartment either by requiring that the largest eigenmode between full and reduced models matches, or by ensuring that the local membrane timescale is the same as in the full model. Both strategies have been found to reproduce the transfer and input response kernels in the full model (symbols match locations in A). **D:** Simulation of the reduced model (dashed, coloured lines) undergoing the BAC-firing protocol, compared to the full model (solid, grey lines). **E:** Code example demonstrating the simplification workflow. NEAT uses the CompartmentFitter class to create reductions based on provided sets of fit locations. The reductions are returned as CompartmentTree class instances

Producing reduced neuron models (Fig. 5A) for efficient network simulations is a major use case of NEAT. Rather than relying on ad-hoc simplification based on e.g. geometry, NEAT’s reduction methodology belongs to a class of algorithm that rely on the electrical response properties of the neuron model. Opposed to prior worked that focused on the dendrite-to-soma transfer resistance (Amsalem et al., 2020), NEAT’s method, as described by (Wybo et al., 2021), uses the full resistance matrix, and therefore accurately captures intra-dendritic interactions. This method has now been extended with the option to incorporate ion concentration dynamics. First, the reduction methodology fits the conductances of the passive model, and then of each ion channel separately, by ensuring that the reduced model approximates the resistance matrix of the full model optimally in the least squares sense (Fig. 5B). To generalize well to the full dynamical range, the reduction methodology performs this fit for up to sixteen expansion points per ion channel at the same time. Once this is done, the concentration dynamics can be computed. NEAT’s aim is to achieve the same ion concentration in the reduced compartment as in the full model at the corresponding site. To achieve this in practice, it has proved sufficient to rescale the total ionic current by a factor computed from the fit of the ion channels that carry the ion under consideration (see supplement). The capacitances are then computed to reproduce the largest eigenmode of the full model, computed from the separation of variables expansion, or by requiring the same membrane time scale as at corresponding locations in the full model. Accuracy of either of these fits can be checked by comparing response kernels in the full and reduced models (Fig. 5C), for which CompartmentFitter provides the plot_kernels() functions. Finally, the reversal potentials of the leak current are used as a compartment-specific free parameter that fits the equilibrium potentials in the reduction to those in the original model at the compartment locations.

The L5PC model by Hay (Hay et al., 2011) that was optimised with an evolutionary algorithm to reproduce backpropagation-activated calcium (BAC) firing, constitutes a challenging test case for the fit methodology. This L5PC model contains fast dendritic sodium channels, slow calcium channels with a high conductance density within the Ca-hotzone (cf. Figure 2B,C), and an h-current increasing exponentially with distance from soma. We ran an identical BAC firing protocol in the original (Fig. 5A, top) and reduced model ((Fig. 5A, bottom) and found qualitatively similar dynamics.

To construct a simplified model in NEAT, it suffices to create a CompartmentFitter, specify a list of locations on the morphology where the compartments should placed, so that they reproduce the dynamics of the full model at those sites, and then call the fit_model() function (Fig. 5E). For technical reasons, this function may append other compartments locations, such as bifurcations that are in between compartment sites (Wybo et al., 2021). As a consequence, the number of compartments in the reduced CompartmentTree may be larger than the number of locations provided to fit_model(). To have a reference to all locations in the reduction, the fit_model() function returns the extended list of compartments together with the reduced CompartmentTree. It should be noted that that the order of the original locations provided to fit_model() will not be scrambled; if *n* locations are provided, the first *n* entries in the returned location list will correspond to the entries in the original location list. Furthermore, each node in the reduced CompartmentTree stores the index of the location in the returned location list that it references, so that they can always be mapped back to the original locations.

Although the simplification approach is demonstrated here with active models, an interesting path towards simplified models is creating reductions of passive morphologies, and subsequently equipping e.g. the somatic compartment with an integrate-and-fire mechanism. In this way, for instance dendritic compartments with NMDA-spikes (Major et al., 2008) can be simulated in a lightweight fashion. In Pastorelli et al. (2025), this approach was used to extend a two-compartment model exhibiting Ca$^{2+}$-spikes with apical and basal compartments with NMDA dynamics.

**Fig. 6:**
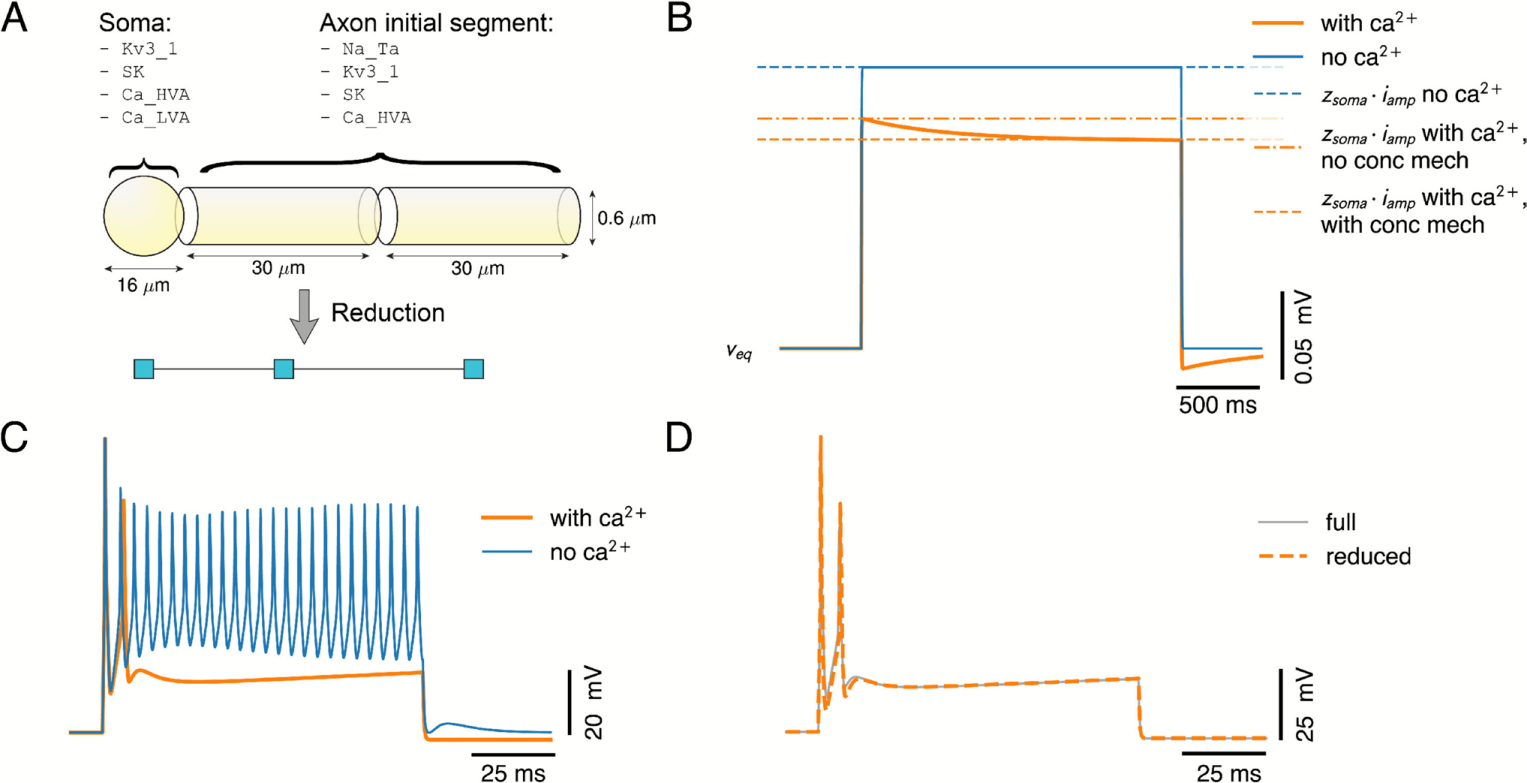
Validation of concentration calculations. **A:** We validate the concentration mechanism in a simplified morphological layout representing a soma and axon initial segment, which, next to the standard K$^{+}$- and Na$^{+}$-channels to generate APs (Kv3_1 resp. Na_Ta), was equipped with Ca$^{2+}$-channels (Ca_HVA and Ca_LVA), Ca$^{2+}$ concentration dynamics, and a Ca$^{2+}$-dependent K$^{+}$-channel (SK). **B:** Validation of the analytical calculations of the somatic input resistance $z_{\text {soma}}$ of the cell. We compare simulated traces with (orange) and without (blue) Ca$^{2+}$ concentration dynamics, with the computed response amplitude (multiplying the $z_{\text {soma}}$ with the current step amplitude $i_{\text {amp}}$), where we linearized *(i)* the neuron model without Ca$^{2+}$ concentration dynamics (blue, dashed), *(ii)* the neuron model without Ca$^{2+}$ concentration dynamics around the expansion point of the model with Ca$^{2+}$ concentration dynamics (orange, dash-dotted), and *(iii)* the neuron model with Ca$^{2+}$ concentration dynamics (orange). **C:** Comparison of the spiking response of the model with (orange) and without (blue) Ca$^{2+}$ concentration dynamics. **D:** Reduction of the model by maintaining one compartment in each cylindrical section (cf. A), demonstrating that the parameters of Ca$^{2+}$ concentration dynamics are appropriately approximated in the reduction

### Validation of the Novel Concentration Dynamics Analysis and Reduction

A novel addition to NEAT is the ability to incorporate concentration mechanisms in the reduction process. Although this is incorporated in the L5PC model (Figs. 2-5), we nevertheless validate this explicitly in a simplified morphological layout representing a soma and axon initial segment (Fig. 6A). First, we confirm that the resistance calculation with GreensTree functions as expected, as the amplitude of the simulated voltage response to a small input current step matches that of the analytically computed linearized response amplitudes (Fig. 6B). Note that we centered these responses at the equilibrium voltage, which is different in both models. This results in a different expansion point for both models ($-82.14$ mV without vs. $-82.67$ mV with Ca$^{2+}$ concentration dynamics), and hence also in different computed response amplitudes when omitting the concentration mechanism from the analytical calculation (dashed blue vs dash-dotted orange in (Fig. 6B). It can further be noted that the computed amplitude without concentration mechanism matches the overshoot, i.e. the response amplitude before the Ca$^{2+}$-adaptation sets in, whereas the computed amplitude with concentration mechanims has incorporated the steady-state influence of the adaptation. Next, we confirm that the Ca$^{2+}$ concentration dynamics in this model strongly affect the spiking response, and through the voltage-dependent K$^{+}$-channel result in adaptation currents that prevent tonic firing (Fig. 6C). Finally, we reduce this axon initial segment to a three-compartment configuration, where the Ca$^{2+}$ dynamics are approximated sufficiently accurate so as to match the voltage and spiking dynamics of the reduced model to the full one (Fig. 6D).

### Simulating Full and Reduced Models in NEURON and NEST

**Fig. 7:**
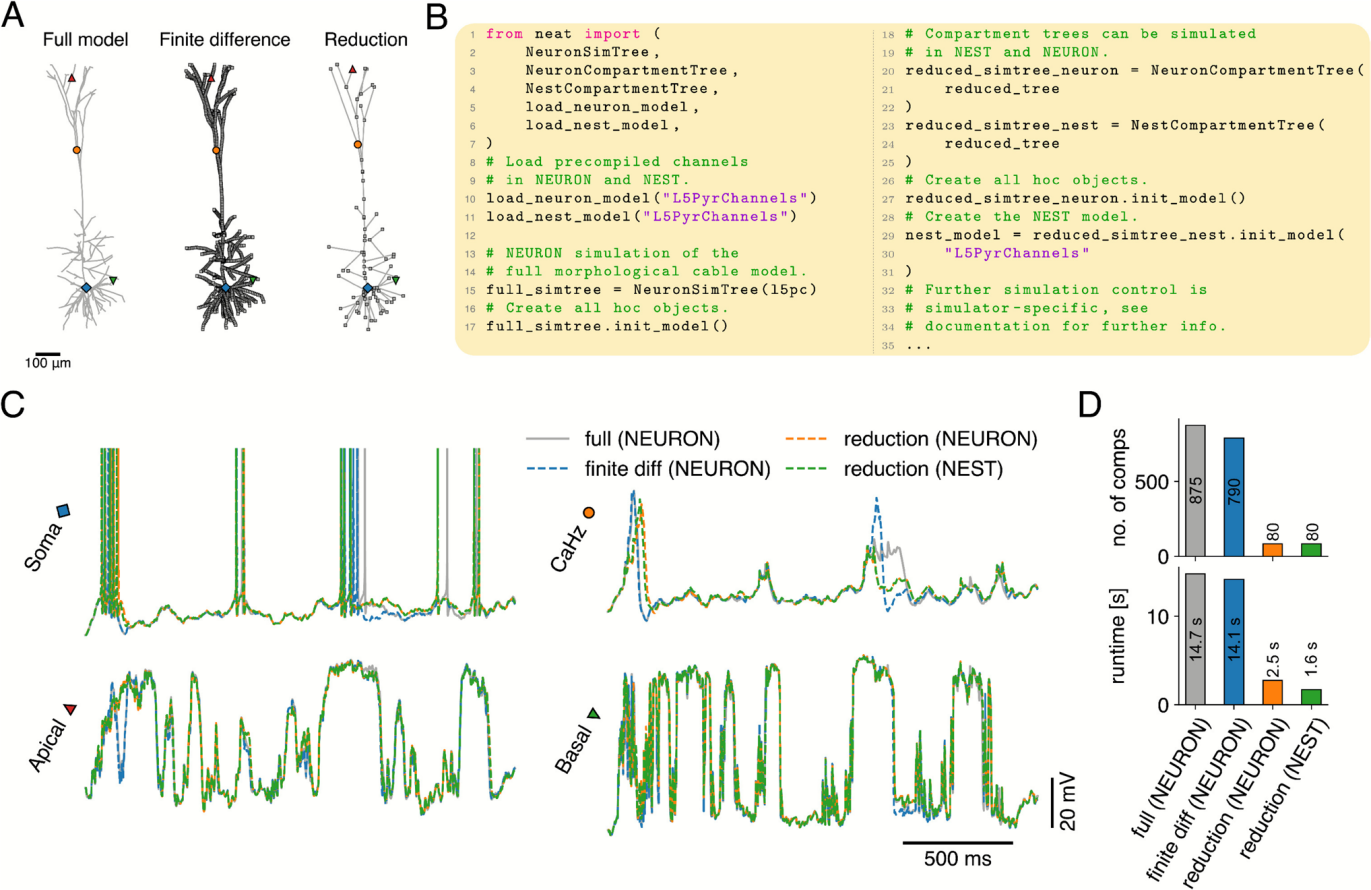
Using NEAT to create simulations in NEURON and NEST. **A:** NEAT can be used to export morphological models – consisting of cylindrical sections (left, full model) – to NEURON, and compartmental models to NEURON and NEST. NEAT provides two ways of obtaining compartmental models from full morphological models: either through the reduction approach (right, reduction) or through NEAT’s finite difference approximation (middle, finite difference). **B:** Code example of exporting models to simulators. NEURON simulations of morphological models – where NEURON internally determines the discretization (i.e. the conversion to a compartmental description) – can be constructed with a NeuronSimTree, whereas NEST resp. NEURON implementations of compartmental models can be constructed with NestCompartmentTree resp. NeuronCompartmentTree. Further simulation control is simulator specific, as each simulator has its own API. **C:** Comparison of voltage dynamics under NEURON’s discretization (grey), NEAT’s finite difference discretization simulated with NEURON (blue), NEAT’s reduction simulated with NEURON (orange), and NEAT’s reduction simulated with NEST (green). Locations as indicated in A. **D:** Number of compartments in the resulting model (top) and the associated runtime for a simulation of 2 s (bottom), simulated on a MacBook M3 Max (NEURON version 8.2.4 and NEST version 3.8.0, both installed following standard procedure as outlined in the respective documentations)

NEAT is not a simulator itself, but a model analysis and reduction tool that exports models to simulators. NEAT can export morphological models to NEURON as connected cylindrical sections, so that NEURON will determine the discretization in compartments internally. NEAT can also export compartmental models to NEURON and NEST. NEAT has two ways of constructing compartmental models for simulation (Fig. 7A): *(*i) through its reduction methodology, and *(ii)* through its implementation of the second order finite difference approximation (cf. Figure S1). In all these cases, the IonChannels that are included in the model need to be compiled first. For this purpose, NEAT provides the neatmodels script, which can be called from the command line (Fig. 1D). Additional mechanisms, such as synaptic receptors, can be included with this script in the model compilation as options. When starting a NEURON or NEST simulation, the compiled model must be loaded first, using the load_neuron_model() or load_nest_model() functions provided in NEAT, so that it is visible to NEURON or NEST (Fig. 7B). Then, the tree classes NeuronSimTree on the one hand, and NeuronCompartmentTree and NestCompartmentTree on the other hand, construct respectively the morphological model in NEURON, or the compartmental model in NEURON or NEST (Fig. 7B).

To compare the compartmentalization methods, reductions and simulators, we have constructed simulations where identical Poisson input trains to glutamatergic (featuring AMPA and NMDA receptors) and GABAergic synapses impinged at equivalent locations (Fig. 7C). We find strong agreement between the intra-dendritic responses in the different models, and also between the overall somatic firing Ca$^{2+}$ spiking behaviour. The precise waveform of the Ca$^{2+}$ spike can differ for different complexity levels and even between NEURON and NEAT’s discretization schemes (cf. supplement) on a case by case basis, highlighting the challenging nature of accurately representing highly non-linear spatio-temporal dynamics. Nevertheless, timing of the associated action potential bursts matched for all cases. As model runtimes scale approximately linearly with the number of compartments, the reduced model – which features approximately ten times fewer compartments than the full models (Fig. 7D, top), has a similarly reduced runtime in NEURON (Fig. 7D, bottom). Interestingly, the NEST implementation achieves a speedup by a factor of almost two over the equivalent NEURON model.

## Discussion and Future Directions

Although NEAT was originally developed as the toolbox implementing the reduction methodology described in Wybo et al. (2021), NEAT has undergone several improvements to broaden its scope and streamline the API. In particular, NEAT’s reduction methodology now admits concentration dynamics, enabling the incorporation of e.g. Ca$^{2+}$-dependent dynamics to elicit dendritic BAC firing. To demonstrate this, we have extended the simplified version of the Hay-model from the original publication – which omitted Ca$^{2+}$-concentration dependent channels – to the full model. NEAT now also allows compartment models to be exported to the NEST simulator for efficient network simulations (Wybo et al., 2025). Finally, NEAT has been extended with performant caching capability, where any type of NEAT-defined model can be cached and receives a hash based on its full state. As a consequence, intermediate computations required for deriving e.g. reduced models are stored automatically, and are loaded seamlessly as starting point for subsequent computational steps. This enables the construction of large scale network models with many different neuron types and simplifications.

Classical analytical methods to compute voltage responses in the dendritic tree can reveal a great deal about dendritic computation, even in the presence of non-linear, voltage-dependent currents. Here, we have highlighted the reason for this: at a short time-scale, dendritic voltage dynamics are dominated by axial currents, which are passive. Therefore, active transmembrane currents can be seen as being integrated by a scaffolding provided by the passive system. This provides an improved understanding of spatiotemporal interactions between dendritic excitation and inhibition (Doron et al., 2017; Gidon and Segev, 2012; Koch et al., 1983; Wybo et al., 2019). Further, it reveals that different loci on the dendritic tree respond preferentially to different input frequencies (Combe et al., 2018; Das et al., 2017; Laudanski et al., 2014; Monai et al., 2010; Ulrich, 2002; Vaidya and Johnston, 2013; Watanabe et al., 2014). However, due to the absence of modern implementations, usage of these algorithms to understand dendritic computation has remained limited, with authors often relying on simplistic morphologies while investigating analytical solutions (e.g. the ‘ball-and-stick’ model, with a linear dendrite) (Bédard and Destexhe, 2008; Pettersen et al., 2014; Schwemmer and Lewis, 2011). Such simple morphologies, however, do not produce the same input resistance profiles from soma to tip as real morphologies, where dendritic segments branch and taper towards the tips, and should therefore be treated with some care. By implementing these algorithms for arbitrary morphologies, NEAT fills this gap in the software toolset of neuroscience.

A powerful usage of these classical analytical methods, in particular Koch’s method to compute quasi-active voltage responses, is to compute large numbers of resistance matrices of the full model, for many expansion points in parallel. This can not be achieved efficiently through simulations, and, as demonstrated by our prior work (Wybo et al., 2021), can be used to constrain the conductance parameters of reduced models. NEAT implements an evolution of this reduction algorithm, which is now extended with the ability to incorporate concentration dynamics in the reduction process. The resulting reductions faithfully approximate full models, but require much less time to simulate, as the simulation cost scales linearly with the number of compartments. NEAT implements functionality to export these reduced models to NEURON (Carnevale and Hines, 2004) and NEST (Espinoza Valverde et al., 2024; Gewaltig and Diesmann, 2007; Linssen et al., 2023). In the future, NEAT plans to support exporting models to other simulators, such as Brian (Stimberg et al., 2019) for models that are easy to adapt, Arbor (Abi Akar et al., 2019; Akar et al., 2023) for biophysically detailed models, or Jaxley (Deistler et al., 2024) to fine-tune parameters.

NEAT exports models to two of the most commonly used simulators in neuroscience, and reads in the most commonly used morphology description format. As such, NEAT is already well embedded in the neuroscientific toolchains. However, NEAT defines its own ion channel format, that is for now also limited to Hodgkin-Huxley type ion channels, as NEAT performs analytical computations using the ion channels. A more general ion channel system is planned for a future version, that is for instance able to read NMODL through the DendroTweaks parser (Makarov et al., 2024), NeuroML (Gleeson et al., 2010), and NESTML (Linssen et al., 2025). Nevertheless, the definition of ion channels is compact and easy to specify. Once they are implemented, they can be compiled into NEURON’s ‘.mod’-files (Carnevale and Hines, 2004) or NESTML models (Blundell et al., 2018; Linssen et al., 2024, 2023), which are in turn compiled into efficient simulation codes by these software packages. All of these steps can be performed through a single terminal command (Fig. 1D). Although NEAT provides model specification tools and has multi-simulator compatibility, it does not aim to be a model specification framework like NeuroML (Gleeson et al., 2010), NeuroConstruct (Gleeson et al., 2007) or PyNN (Davison, 2009). Rather, NEAT aims to increase its compatibility with these tools in future developments.

Reduced models of dendritic computation can be grouped broadly speaking in two categories: *(i)* top-down, where the model is built up to capture a particular dendritic computation, in a manner that is as simple as possible (Naud et al., 2014; Pagkalos et al., 2023; Pastorelli et al., 2025; Quaresima et al., 2022; Zhang et al., 2024), and *(ii)* bottom-up, were starting from a full morphology, a mathematical simplification procedure is applied to arrive at a simpler model (Amsalem et al., 2020; Hendrickson et al., 2011; Makarov et al., 2024; Marasco et al., 2013). NEAT’s simplification algorithm can be employed in both cases (Pastorelli et al., 2025; Wybo et al., 2021), as it provides the freedom to focus on specific computations by retaining appropriate sets of locations on the morphology, while accurately capturing both intra-dendritic and somato-dendritic interactions. In modern approaches, it is often desirable to capture multiple dendritic computations in a realistic fashion (Pagkalos et al., 2023). NEAT was used, for instance, to show that a two-compartment adaptive exponential integrate-and-fire model that exhibits BAC-firing, can be extended with NEAT to have further apical and basal compartments that support NMDA spikes (Pastorelli et al., 2025).

Taken together, NEAT fills a gap in the neuroscientific software toolset by on the one hand allowing the study of single neuron properties in great detail, through its analytical algorithms and model export to NEURON functionality, and on the other hand by its functionality to embed simplified versions of the *same* neuron model in large-scale networks, through its export to NEST functionality. As such, NEAT helps to bridge a long-lasting divide in computational neuroscience, where – with some notable exceptions (Billeh et al., 2020; Markram et al., 2015; Migliore et al., 2014) – research is generally focused on one or a few highly detailed models, or on large networks of highly simplified point-neurons.

## Availability and Support

NEAT can be installed through pip (https://pypi.org/project/nest-neat/) or from the source repository (https://github.com/nest/NEAT). Further documentation on NEAT can be found at https://neatdend.readthedocs.io/. The full code to reproduce the figures from this manuscript can be found at https://jugit.fz-juelich.de/w.wybo/NEATPaper. NEAT is part of the NEST initiative, which means users can seek support through the NEST mailing list (users@nest-simulator.org), the frequent hackathons and tutorial sessions at conferences, and the yearly NEST conference.

## Supplementary Information

Below is the link to the electronic supplementary material.

Supplementary file 1 (pdf 787 KB)

## Data Availability

No datasets were generated or analysed during the current study.
