# Supplementary material for: The Neural Analysis Toolkit Unifies Semi-Analytical Techniques to Simplify, Understand, and Simulate Dendrites
Source: Neuroinformatics. 2026 Apr 14;24(2):21. doi: 10.1007/s12021-025-09766-x (PMC13079553; doi:10.1007/s12021-025-09766-x)
Supplement: Supplementary file 1 — (pdf 787 KB) [file 12021_2025_9766_MOESM1_ESM.pdf]

Supplementary information

S1 Mathematical formulation of morphological neuron models

S1.1 Neurons as systems of coupled cables

In the morphological tree classes in NEAT, nodes are taken to represent cylindrical sections of neurite, which are connected at their proximal end to the cylindrical section associated with their parent node, whereas at their distal end they connect to the cylindrical sections associated with their child nodes. On such a cylindrical section associated with node n, membrane voltage is modeled by the cable equation (Tuckwell 1988), which was first applied to dendrites by Rall (Rall 1959, 1960, 1962). With the inclusion of active channels and synaptic inputs, this equation has the following form:

$$c_m \frac{\partial v_n}{\partial t}(x,t) - \frac{a}{2r_a}\frac{\partial^2 v_n}{\partial x^2}(x,t) + g_l\,(v_n(x,t) - e_l) + \sum_c i_{cn}(v_n(x,t), \mathbf{y}_{cn}(x,t)) = \sum_i i_{in}(t, v_n(x_i,t))\,\delta(x - x_i), \tag{1}$$

where c_m, r_a, g_l, and e_l denote, respectively, the membrane capacitance, the the axial resistance, the leak conductance and the leak reversal, a denotes the radius of the dendritic branch, i_c the current contribution of a channel type c and i_i the a-priori arbitrary input current at location x_i. The ion channel current i_c can depend non-linearly on the voltage and a number of state-variables $\mathbf{y}_{cn}$. Note that the parameters are all specified on a per-node basis, but for notational clarity the node index was not made explicit. At the moment, NEAT only accommodates channel currents of the Hodgkin-Huxley type (Hodgkin and Huxley 1952):

$$i_c(v(\xi,t), \mathbf{y}_c(\xi,t)) = \overline{g}_c\, o_c(\mathbf{y}_c(\xi,t))\,(v(\xi,t) - e_c), \tag{2}$$

so that the channel current depends on a maximal conductance density $\overline{g}_c$, a driving force $(v(x,t) - e_c)$ – with e_c the channel's reversal potential – and $o_c(\mathbf{y}_c(\xi,t))$ the channel's open probability, which depends on a number of state variables $\mathbf{y}_c(\xi,t) = (y_{c1}(\xi,t), \ldots, y_{cK}(\xi,t))$ that evolve according to

$$\dot{y}_{ck}(\xi,t) = \frac{y_{ck,\infty}(v(\xi,t)) - y_{ck}(\xi,t)}{\tau_{ck}(v(\xi,t))},\ k = 1,\ldots,K, \tag{3}$$

with $\tau_{ck}(v)$ and $y_{ck,\infty}(v)$ functions that depend on the channel type. In NEAT, all parameters are stored on a per-node basis. Hence, each cylindrical section has spatially uniform parameters, but they can have different values for each node. By consequence, spatially non-uniform physiological parameters are represented in a piecewise constant manner, and the resolution of this approximation corresponds to the points from the underlying .swc-file. Should this resolution be insufficient, the tree can be resampled through `MorphTree.create_new_tree()`. Note furthermore that NEAT only considers the left-hand side of equation (1) in its analysis tools. A-priori arbitrary sets of inputs can be added in the models that are exported for simulation.

Voltage dynamics are constrained by boundary conditions (Tuckwell 1988)). When tree nodes are leafs (i.e. they have no child nodes, and represent the most distal segments of neurite), we assume a sealed-end boundary condition (no longitudinal current flow):

$$\left.\frac{\partial v_n}{\partial x}(x,t)\right|_{x=L_n} = 0. \tag{4}$$

Where two or more cylinders join together, the boundary conditions are given by requiring the

equality of the membrane potential:

$$v_n(x,t)|_{x=L_n} = v_m(x,t)|_{x=0}\,, \quad \forall m \in \mathcal{C}_n, \tag{5}$$

and conservation of longitudinal current flow:

$$\frac{a_n^2}{r_{a,n}} \frac{\partial v_n}{\partial x}(x,t)\bigg|_{x=L_n} = \sum_{m\in\mathcal{C}_n} \frac{a_m^2}{r_{a,m}} \frac{\partial v_m}{\partial x}(x,t)\bigg|_{x=0}\,, \tag{6}$$

where $\mathcal{C}_n$ denotes the set of child nodes of node n. Finally, at the soma, we apply the lumped-soma boundary condition. To this purpose, the root node of the tree, which in NEAT is assumed to implement a spherical soma of radius a_s, is modeled electrically as a single compartment with voltage v_s. The boundary conditions then stipulate equality of the membrane potential:

$$v_s(t) = v_m(x,t)|_{x=0}\,, \quad \forall m \in \mathcal{C}_s, \tag{7}$$

and require that the total current flow through the somatic membrane must equal the axial current flow through the branches emanating from the soma:

$$\begin{aligned} c_s \frac{\mathrm{d}v_s}{\mathrm{d}t}(t) + g_{l,s}(v_s(t) - e_{l,s}) + \sum_c i_c(\mathbf{y}_{c,s}(t), v_s(t)) \\ = \frac{1}{4\pi a_s^2} \sum_{m\in\mathcal{C}_s} \frac{\pi a_m^2}{4 r_{a,m}} \frac{\partial v_m}{\partial x}(x,t)\bigg|_{x=0}. \end{aligned} \tag{8}$$

Here, somatic voltage and parameters are denoted with the subscript s.

S1.2 The quasi-active approximation for computing linearized neural dynamics

The system of equations described above can be linearized around an a-priori arbitrary expansion point $v(\xi,t) = v_0(\xi) + \delta v(\xi,t)$ for voltage and $\mathbf{y}_c(\xi,t) = \mathbf{y}_{c,0}(\xi) + \delta\mathbf{y}_c(\xi,t)$ for ion channel state variables (Mauro et al. 1970). In NEAT, this expansion point can be specified on a per-node basis, and hence is constant for each cylindrical section; NEAT implicitely assumes the approximation $v_0(\xi) \equiv v_{0,n}(x) \approx v_{0n}$ and $\mathbf{y}_{c,0}(\xi) \equiv \mathbf{y}_{c,0,n}(x) \approx \mathbf{y}_{c,0,n}$. Linearizing ion channel currents then yields a quasi-active description (Koch 1998) of the neuron model:

$$\begin{aligned} i_{c,\mathrm{lin}} =& \overline{g}_c(e_c - v_0) \sum_k \frac{\partial o_c}{\partial y_{c,k}}\bigg|_{y_{c,k}(\xi,t)=y_{c,0,k}} \delta y_{c,k}(\xi,t) \\ &+ \overline{g}_c o_c(\mathbf{y}_{c,0})\, \delta v(\xi,t), \end{aligned} \tag{9}$$

with

$$\begin{aligned} \dot{\delta y}_{c,k}(\xi,t) =& \frac{\mathrm{d}}{\mathrm{d}v}\left(\frac{y_{c,k,\mathrm{inf}}}{\tau_{c,k}}\right)_{v=v_0} \delta v(\xi,t) \\ &- \frac{1}{\tau_{c,k}(v_0)} \delta y_{c,k}(\xi,t), \end{aligned} \tag{10}$$

where all derivatives, as well as $\tau_{c,j}$, are evaluated at the expansion point of the state variables. If the expansion point given by $v_0(\xi)$ and $\mathbf{y}_{c,0}(\xi)$ is stable, then $\delta v(\xi,t)$ and $\delta\mathbf{y}_c(\xi,t)$ will model the responses to transient input perturbations that are small enough so that the linear approximation around the expansion point is valid. A natural special case of this is the situation where the expansion point is the equilibrium of the neuron in the absence of external inputs. The linearization then models responses to small inputs. This quasi-active approximation is assumed by the `GreensTree` and the `GreensTreeTime` in their implementation of the Green's function calculation.

S1.3 The membrane leak approximation for computing passified neural dynamics

A further approximation of (1) can be obtained by replacing the dynamic channel open probabilities $o(\mathbf{y}_{c,n}(x,t))$ with their value at the expansion point $v_{0,n}$, $\mathbf{y}_{c,0,n}$, so that the channel conductance $\overline{g}_c\, o_c(\mathbf{y}_{c,0,n})$ is a constant which is added to the leak:

$$g_{l,n}\,(v(\xi,t) - e_{l,n}) \to g'_{l,n}\,(v(\xi,t) - e'_{l,n}) \tag{11}$$

where

$$\begin{aligned} g'_{l,n} &= g_{l,n} + \sum_c \overline{g}_{c,n}\, o_c(\mathbf{y}_{c,0,n}), \\ e'_{l,n} &= \frac{g_{l,n}\, e_{l,n} + \sum_c \overline{g}_{c,n}\, o_c(\mathbf{y}_{c,0,n})\, e_c}{g'_{l,n}}. \end{aligned} \tag{12}$$

In doing so, the neuron model is converted to a passive one. Despite the strong reduction, this approximation still provides useful information. In particular, the longitudinal spread of the voltage is dominated by the axial current, and therefore morphological properties such as the radii of the cylinders. By consequence, this 'passified' neuron model is remarkably accurate in reproducing the effective compartmentalization (Wybo et al. 2019) of the original model (cf. Fig 4). Furthermore, NEAT's `SOVTree`, which implements the seperation-of-variables solution, assumes this description, as this solution has only been derived for passive neuron models. Finally, reducing certain sections of the dendritic tree to the passive approximation may yield sizeable efficiency gains in simulations, and could be justified with respect to certain computations. For instance, local subunits arising through the nonlinearity of the NMDA-receptor can be reproduced in such an approximation (Wybo et al. 2019). To passify models or certain groups of nodes, NEAT's `PhysTree` and derived classes provide the `as_passive_membrane()` function.

S2 The Green's function of a morphological neuron model

In essence, the Green's function $Z(\xi, \xi_i; t)$ is the solution of the system of linearized, coupled cable equations to a delta pulse input current $\delta_{nm}\delta(x - x_i)\delta(t)$ occurring at a certain site $\xi_i = (m, \frac{x_i}{L_m})$ on the neuron at time $t = 0$ (Tuckwell 1988; Koch 1998). From this, the solution to a general input current perturbation $\delta i(x'_m, t)$ can be constructed through convolution:

$$v(\xi,t)=\sum_m \int_0^1 \mathrm{d}\overline{x} \int_0^\infty \mathrm{d}\tau [Z(\xi,\xi_i,\tau)\delta i_m(\xi_i,t-\tau)]_{\xi=(m,\overline{x})}. \tag{13}$$

Here the sum runs over all nodes m, and the spatial integral integrates over all the associated cylinders. In nearly all practical use cases, the inputs occur at a discrete set of point-like locations in space (e.g. synaptic receptor sites or electrode current injection sites), which will referred to as input sites $\{\xi_i\}$ for notational simplicity. Mathematically, the input is thus a combination of spatial delta functions, allowing us to reduce the compound spatial sum and integral in (13) to a simple sum, i.e.

$$v(\xi, t) = \sum_i \int_{\tau>0} \mathrm{d}\tau \, Z(\xi, \xi_i, \tau) \, \delta i(\xi_i, t - \tau). \tag{14}$$

The significance of the Green's function is that it encapsulates all effects induced by the morphology in a single function, and therefore contains much information about the neuron's response properties. For instance, input or transfer resistances, measured in response to e.g. DC current steps, are given by the integral $Z(\xi, \xi') = \int_{\tau>0} \mathrm{d}\tau \, Z(\xi, \xi', \tau)$. The Fourier transform of the Green's function, $\tilde{Z}(\xi, \xi', \omega)$, yields the input or transfer impedances, i.e. the voltage response amplitude and phase at a site ξ in response to a sinusoidal input current at site ξ'. Furthermore, $Z(\xi, \xi', t)$ is also known as the impulse response kernel, and contains information on the response time-scales induced by the neuronal morphology. Finally, constructing simplified models that approximate parts of $Z(\xi, \xi', \tau)$ as well as possible has proven to be a powerful method of reducing complex neuron models (Wybo et al. 2021).

S2.1 Computing the Green's function in the frequency domain: input and transfer impedances

By Fourier transforming (14) to the frequency domain, the temporal convolution becomes a multiplication:

$$\tilde{v}(\xi, \omega) = \sum_i \tilde{Z}(\xi, \xi_i, \omega) \, \tilde{\delta i}(\xi_i, \omega), \tag{15}$$

which allows for the straightforward computation of the voltage response amplitude and phase in response to specific input currents. By analysing the impedance $\tilde{Z}(\xi, \xi_i, \omega)$, it can be shown that while passive models are always low-pass filters of the input current, ion channels can cause neurons to become band-pass filters, preferentially responding to certain frequency bands (Ulrich 2002; Monai et al. 2010; Vaidya and Johnston 2013; Laudanski et al. 2014; Watanabe et al. 2014; Das et al. 2017; Combe et al. 2018). Furthermore, the specific preferred frequency depends on the location on the morphology (Laudanski et al. 2014). Koch's algorithm (Koch and Poggio 1985), based on graphical rules derived by Butz and

Cowan (Butz and Cowan 1974), computes these impedances exactly on tree graphs. If the graph were to contain loops, which might be induced if the impedance needs to be calculated for multiple neurons connected by gap junctions, Abbott's sum-over-trips approach provides an approximate solution (Cao and Abbott 1993; Coombes et al. 2007; Timofeeva and Coombes 2014).

NEAT implements Koch's algorithm through the `GreensTree` class, which is briefly sketched here. First, it should be noted that Fourier transforming the linearized neuron model yields a coupled system of 2nd-order ODEs, as temporal derivatives are replace by multiplication with $i\omega$ – where i is the imaginary unit. For the linearized cable equation, we obtain

$$-\frac{a}{2r_a}\frac{\partial^2\tilde{\delta v}}{\partial x^2}(x,\omega)+i\omega\,\tilde{\delta v}(x,\omega)+g_l\,\tilde{\delta v}(x,\omega)$$
$$+\,\overline{g}_c(e_c-v_0)\sum_k \left.\frac{\partial o_c}{\partial y_{c,k}}\right|_{y_{c,k}(x,t)=y_{c,0,k}}\tilde{\delta y}_{c,k}(x,\omega)+\overline{g}_c o_c(\mathbf{y}_{c,0})\,\tilde{\delta v}(x,\omega)=\sum_{i=1}^{n}\tilde{\delta i}_i(\omega)\,\delta(x-x_i). \tag{16}$$

The Fourier transformed ion channel state variables can then be eliminated from this equation, by rearranging the Fourier transforms of their quasi-active evolution equations (10):

$$\tilde{\delta y}_{c,k}(x,\omega)=\frac{\frac{\mathrm{d}}{\mathrm{d}v}\left(\frac{y_{c,k,\mathrm{inf}}}{\tau_{c,k}}\right)_{v=v_0}}{i\omega+\frac{1}{\tau_{c,k}(v_0)}}\,\tilde{\delta v}(x,\omega), \tag{17}$$

yielding

$$\frac{\partial^2\tilde{\delta v}}{\partial x^2}(x,\omega)-\frac{1}{\lambda(\omega)^2}\tilde{\delta v}(x,\omega)=-\sum_{i=1}^{n}\tilde{\delta i}_i(\omega)\,\delta(x-x_i), \tag{18}$$

where the frequency-dependent length constant $\lambda(\omega)$ is given by:

$$\lambda(\omega)\equiv\sqrt{\frac{2r_a}{a}\left(i\omega+g_l+\sum_c\overline{g}_c\left[(e_c-v_0)\sum_k\frac{\left.\frac{\partial o_c}{\partial y_{c,k}}\right|_{y_{c,k}(x,t)=y_{c,0,k}}\frac{\mathrm{d}}{\mathrm{d}v}\left(\frac{y_{c,k,\mathrm{inf}}}{\tau_{c,k}}\right)_{v=v_0}}{i\omega+\frac{1}{\tau_{c,k}(v_0)}}+o_c(\mathbf{y}_{c,0})\right]\right)^{-1}}. \tag{19}$$

In the remainder of this section we will drop the dependence on ω for notational clarity.

It can be seen that $\cosh(x/\lambda)$ and $\sinh(x/\lambda)$ are two linearly independent fundamental solutions of the homogeneous version of (18), i.e. where the input (right-hand side) is zero. Under homogeneous boundary conditions

$$\left.\frac{\partial\tilde{\delta v}}{\partial x}(x)\right|_{x=0,L}+\beta\,\tilde{\delta v}(x)\Big|_{x=0,L}=0, \tag{20}$$

the solution to a delta-pulse input at x_i is:

$$\tilde{\delta v}(x)=\begin{cases}\frac{p(x)q(x_i)}{w(p,q,x_i)}, & \text{for } x<x_i\\ \frac{p(x_i)q(x)}{w(p,q,x_i)}, & \text{for } x>x_i,\end{cases} \tag{21}$$

with p resp. q linear combinations of the fundamental solutions that satisfy the homogeous boundary condition resp. at $x=0$ and at $x=L$, and $w(p,q,x)=p(x)\,q'(x)-q(x)\,p'(x)$ the Wronskian of the solutions (Stakgold 1967).

The issue is that at the junctions between coupled cylinders, the boundary conditions (5) and (6) are not homogenuous (and neither is

the lumped-soma boundary condition). However, starting from the leaf cylinders, where it can be seen that the sealed-end condition (4) is homogenuous, homogenuous boundary conditions can be constructed for the whole tree. Let $q_m(x) = \cosh(\frac{x-L_m}{\lambda_m})$ be the solution on such a leaf cylinder m that satisfies (4) on the sealed end. Every non-trivial solution on this cylinder, in the absence of direct input to the cylinder, will be of the form $\alpha_m \, q_m(x)$ for some factor α_m. At the junction with the parent cylinder n, we get that $\alpha_m = \frac{v_n(x,t)|_{x=L_n}}{q_m(x=0)}$ from (5), which leads to a constraint on the derivative:

$$\frac{\partial v_m}{\partial x}(x,t)\bigg|_{x=0} = \frac{v_n(x,t)|_{x=L_n}}{q_m(x=0)} \, q'(x=0). \quad (22)$$

Substituting this in (6) then yields:

$$\frac{\partial v_n}{\partial x}(x,t)\bigg|_{x=L_n} - \beta_n^{\text{dist}} \; v_n(x,t)|_{x=L_n} = 0, \quad (23)$$

where $\beta_n^{\text{dist}} = \frac{r_{a,n}}{a_n^2} \frac{a_m^2}{r_{a,m}} \frac{q_m'(x=0)}{q_m(x=0)}$. It can be seen that this is precisely the homogeneous boundary condition we were after. In the parent cylinder n, we can then again construct a homogeneous solution $q_n(x)$ from the fundamental solutions $\cosh(x/\lambda_n)$ and $\sinh(x/\lambda_n)$, that satisfies the constructed boundary condition (23). Note that this scheme can readily be extended to a junction where the parent cylinder has more than one child node, where we obtain for β_n^{dist}:

$$\beta_n^{\text{dist}} = \frac{r_{a,n}}{a_n^2} \sum_{m \in \mathcal{C}_n} \frac{a_m^2}{r_{a,m}} \frac{q_m'(x=0)}{q_m(x=0)}. \quad (24)$$

This scheme can then be applied recursively through the tree graph. When the recursion arrives at the soma, working out the lumped soma boundary condition in the same way results in a homogenuous boundary condition (20) on the proximal end of each of the child cylinders of the soma. Continuing the recursive scheme, but now towards the leafs, then yields homogenuous boundary conditions at the proximal ends for each cylinder (for which we call the associated factors β_n^{prox}. When the `GreensTree.set_impedance()` function is called, NEAT computes the factors β_n^{prox} and β_n^{dist} for each node n through this recursive algorithm.

Finally, to compute the Green's function in response to a delta pulse at location x_i on node n, NEAT uses (21), where $p_n(x)$ and $q_n(x)$ are computed from the homogenuous boundary conditions (20) on proximal resp. distal ends, using the stored factors β_n^{prox} resp. β_n^{dist}. On the child cylinders $m \in \mathcal{C}_n$, the solution is then of the form $\alpha_m \, q_m(x)$, with $q_m(x)$ computed from the fundamental solutions by satisfying the distal homogenuous boundary condition (i.e. computed from β_n^{dist}). The continuity of voltage condition (5) at the junction between cylinder n and cylinder m is then used to compute α_m, and this scheme is repeated recursively until the dendritic tips are reached. Similarly, on the proximal end – in the parent cylinder k we use the solution $\alpha_k p_k(x)$ which satisfied the proximal homogenuous boundary condition (i.e. computed from β_k^{prox}), where the prefactor α_k is again computed from the continuity of voltage condition, now at the junction between cylinders k and n. This scheme is then repeated recursively until the soma is reached. Note that in practice, the Green's function is always evaluated between a discrete set of locations, e.g. through calling `calc_zf()` or `calc_impedance_matrix()`, and the recursion will only be applied to the direct path between the input location and the target location(s).

S2.2 Inverse Fourier transform algorithms to compute the Green's function in the time domain

While the `GreensTree` computes the impedances exactly in the frequency domain, time-domain impulse response kernels are obtained in NEAT with `GreensTreeTime`, which implements algorithms that compute the inverse Fourier transform

$$Z(\xi,\xi',t) = \mathcal{F}^{-1}[\tilde{Z}(\xi,\xi',\omega)] \equiv \int_{-\infty}^{\infty} d\omega e^{-i\omega t} \tilde{Z}(\xi,\xi',\omega). \quad (25)$$

Computing this quadrature accurately is not a straightforward problem: on the one hand, these kernels need to be accurate on submillisecond times-scales, to capture voltage transfer between nearby locations, while on the other hand, they need to be accurate on long time-scales (10-100 ms), to capture the voltage decay through the

membrane. This means that the quadrature must be computed precisely for small frequencies (long time-scale accuracy), but that it also must incorporate very large frequencies (for short time-scale accuracy). To that purpose, NEAT by default defines an integration grid $\{\omega_i\}$ that is composed of 100 equispaced frequency values between -10 and 10 Hz, and a log-spaced frequency grid containing 200 values from 10 to 10^7Hz (and similarly from -10^7 to -10 Hz. Note that with this grid, $\tilde{Z}(\xi, \xi_i, \omega)$ was empirically found to always change smoothly between grid points. However, computing the quadrature (25) naively would still be highly inaccurate, as $e^{-i\omega t}$ might oscillate strongly as a function of t, with a period smaller than the distance between integration grid points. To resolve this issue, NEAT linearly interpolates $\tilde{Z}(\xi, \xi_i, \omega)$ between grid points:

$$Z(\xi,\xi',t)=\sum_{i=1}^{K-1}\int_{\omega_i}^{\omega_{i+1}} \mathrm{d}\omega e^{-i\omega t} \left[\tilde{Z}(\xi,\xi',\omega_i)+\frac{\tilde{Z}(\xi,\xi',\omega_{i+1})-\tilde{Z}(\xi,\xi',\omega_i)}{\omega_{i+1}-\omega_i}(\omega-\omega_i)\right], \tag{26}$$

with K the number of grid points, and computes the resulting integrals $\int_{\omega_i}^{\omega_{i+1}} \mathrm{d}\omega\, e^{-i\omega t}$ and $\int_{\omega_i}^{\omega_{i+1}} \mathrm{d}\omega\, e^{-i\omega t}$ exactly. Finally, these integrals are precomputed for each desired t-value at which the impulse response kernel is to be evaluated, and are arranged in a matrix so that the whole quadrature is recast as a matrix-vector product with the vector $(\tilde{Z}(\xi, \xi', \omega_1), ..., \tilde{Z}(\xi, \xi', \omega_K))$.

The quadruature-based approach works well for transfer kernels, where $\xi \neq \xi'$. This is the case because transfer kernels are continuous everywhere (in particular at $t = 0$, as they are identically zero for $t < 0$ (implementing causality), are zero at $t = 0$ (it takes a non-zero amount of time for a current injection at a site ξ' to cause a voltage perturbation at site $\xi \neq \xi'$, and then reach nonzero values for $t > 0$. This means that the frequency domain transfer impedance decays to zero for $\omega \to \infty$, and in particular that $|\tilde{Z}(\xi, \xi_i, \omega)|$ is vanishingly small at the edges of the quadrature grid (i.e. $\omega = -10^7$ and 10^7Hz). For input kernels however, where $\xi = \xi'$, $Z(\xi, \xi, t)$ jumps discontinuously to a non-zero value at $t = 0$ (a current injection at site ξ will instantaneously cause a voltage perturbation at that site), the associated input impedance will contain all frequencies, and converge to a non-zero value for $\omega \to \infty$. Concretely, this means that any quadrature approach will create an artificial cutoff frequency, in turn inducing spurious oscillations in the time domain kernel. To resolve this issue, it can be noted that input kernels decay from their initial value. This decay can be approximated accurately as a superposition of exponentials:

$$Z(\xi, \xi, t) \approx \begin{cases} \sum_{k=1}^{K} \gamma_k\, e^{-\alpha_k t}, & \text{for } t \geqslant 0 \\ 0, & \text{for } t < 0, \end{cases} \tag{27}$$

with $\mathrm{Re}(\alpha_k) > 0$. By default, NEAT sets $K = 20$, as this was empirically found to be a good compromise between speed and accuracy. In the frequency domain, such a representation is of the form:

$$\tilde{Z}(\xi, \xi, \omega) \approx \sum_{k=1}^{K} \frac{\gamma_k}{i\omega + \alpha_k}. \tag{28}$$

NEAT implements the vector fitting algorithm (Gustavsen and Semlyen 1998, 1999) to approximate input impedances in the frequency domain in this way. For the desired t-values, the input kernels are then computed according to (27).

S2.3 The Green's function as a superposition of exponentials: the separation of variables solution

The Green's function can also be computed directly as a superposition of exponentials

$$Z(\xi, \xi', t) = \sum_{k=1}^{\infty} \phi_k(\xi)\, \phi_k(\xi')\, e^{-\alpha_k t}, \text{ with } \alpha_k > 0 \tag{29}$$

through the separation of variables expansion, which yields an infinite series of response timescales $1/\alpha_k$ and associated spatial profiles $\phi_k(\xi)$ (Holmes et al. 1992; Major et al. 1993b,a; Major 1993; Major and Evans 1994). This formulation has as advantage that the voltage response can be decomposed in a number of distinct modes, each with an associated timescale, which can be seen

from substituting (29) in (14):

$$v(\xi,t)=\sum_k \phi_k(\xi)\rho_k(t),$$
$$\text{with } \rho_k(t)=\sum_i \phi_k(\xi_i)\int_0^\infty \mathrm{d}\tau\, e^{-\alpha_k \tau}\,\delta i(\xi_i,t-\tau). \tag{30}$$

So far, the separation of variables expansion has only been formulated for passive neuron models. In NEAT, this means that it is automatically applied to the passified version (cf. (11), (12)) of (4). First, solutions to the homogeneous problem are computed. On a single cylinder, the passified cable equation has the form (1):

$$\lambda^2 \frac{\partial^2 \delta v}{\partial x^2}(x,t) - \tau_m \frac{\partial \delta v}{\partial t}(x,t) - \delta v(x,t) = 0, \tag{31}$$

with $\lambda = \sqrt{2 r_a g_l' / a}^{-1}$ the length constant and $\tau_m = c_m / g_l'$ the membrane time constant. In the separation of variables expansion, solutions to (31) are proposed of the form:

$$v(x,t) = \phi(x)\, e^{-\alpha t}. \tag{32}$$

Substituting this in (31) yields:

$$\phi(x) = \kappa \left[\cos\left(\frac{\nu\,(L-x)}{\lambda}\right) + \mu \sin\left(\frac{\nu\,(L-x)}{\lambda}\right)\right], \tag{33}$$

where κ and μ are parameters to be determined from the boundary conditions, and $\nu = \sqrt{\alpha\,\tau_m - 1}$. Major's algorithm then determines the parameters μ in a recursive procedure, starting from the distal tips. Here, the sealed end condition holds, leading to $\mu = 0$. Applying the continuity of voltage condition (5) at junctions between nodes then yields:

$$\kappa_n = \kappa_m \left[\cos\left(\frac{\nu_m L_m}{\lambda_m}\right) + \mu_m \sin\left(\frac{\nu_m L_m}{\lambda_m}\right)\right], m \in \mathcal{C}_n, \tag{34}$$

where we have reintroduced the indices n for parent cylinder en $m \in \mathcal{C}_n$ for the child cylinders. The conservation of current condition (6) stipulates:

$$-\kappa_n \mu_n \frac{\nu_n}{\lambda_n}\frac{a_n^2}{r_{a,n}} = \sum_{m\in\mathcal{C}_n} \kappa_m \frac{\nu_m}{\lambda_m}\frac{a_m^2}{r_{amn}} \left[\sin\left(\frac{\nu_m L_m}{\lambda_m}\right) - \mu_m \cos\left(\frac{\nu_m L_m}{\lambda_m}\right)\right]. \tag{35}$$

From this, the ratio κ_m/κ_n is eliminated, to find a recursive relation that solves for μ_n throughout the tree:

$$\mu_n = \sum_{m\in\mathcal{C}_n} \frac{\frac{\nu_m}{\lambda_m}\frac{a_m^2}{r_{amn}}}{\frac{\nu_n}{\lambda_n}\frac{a_n^2}{r_{a,n}}} \left[\frac{\mu_m \cos\left(\frac{\nu_m L_m}{\lambda_m}\right) - \sin\left(\frac{\nu_m L_m}{\lambda_m}\right)}{\cos\left(\frac{\nu_m L_m}{\lambda_m}\right) + \mu_m \sin\left(\frac{\nu_m L}{\lambda_m}\right)}\right], \tag{36}$$

where we note that these analytical expressions depend on the yet unknown constant α, since ν depends on α. The somatic boundary condition (8) then results in:

$$\alpha\, c_s - g_{l,s} = \frac{1}{4\, a_s^2} \sum_{m\in\mathcal{C}_s} \frac{a_m^2}{4 r_{a,m}} \frac{\nu_m}{\lambda_m} \left[\frac{\mu_m \cos\left(\frac{\nu_m L_m}{\lambda_m}\right) - \sin\left(\frac{\nu_m L_m}{\lambda_m}\right)}{\cos\left(\frac{\nu_m L_m}{\lambda_m}\right) + \mu_m \sin\left(\frac{\nu_m L_m}{\lambda_m}\right)}\right]. \tag{37}$$

Thus, equations (36) and (37) define a recursive transcendental equation, whose solutions are the constants α_k in (29). NEAT adapts Major's approach (Major et al. 1993a) to solve this equation for the α_k's, but replaces the zero finding algorithm with complex analysis techniques based on contour integration (Kravanja and Van Barel 2000).

It can be noted that the ratios between all k_n and k_m ($m \in \mathcal{C}_n$) are fixed by (34), and therefore that they are determined up to a global constant. Major (Major et al. 1993a) shows that for a delta-pulse input at a location ξ', this global constant is $\gamma_k \phi_k(\xi')$, with γ_k a factor that is analytically computable once α_k is known. Therefore, it follows that:

$$Z(\xi,\xi',t) = \sum_{k=1}^{\infty} \gamma_k \phi_k(\xi)\,\phi_k(\xi')\, e^{-\alpha_k t}. \tag{38}$$

NEAT adopts the convention $\sqrt{\gamma_k}\phi_k \to \phi_k$ when returning these functions (for instance through `SOVTree.get_important_modes()`), thus yielding (29).

S3 The mathematics underlying compartmental neuron models

S3.1 Neurons as systems of coupled compartments

A compartmental neuron model is governed by a system of equations of the form

$$\begin{aligned} c_m\,\dot{v}_i(t) &+ \sum_{j\in\mathcal{N}_i} g_{ij}\,(v_i(t)-v_j(t)) + g_l\,(v_i(t)-e_l) \\ &+ \sum_c i_c(v_i(t), \mathbf{y}_{ci}(t)) \\ &= \sum_{i=1}^{n} i_i(t, v_i(t))\,\delta(x-x_i),\ i=1,\ldots,N_c. \end{aligned} \tag{39}$$

Here, the spatial coordinate $\xi = (n, \overline{x})$ is replaced by a compartment index i, and parameters such as capacitance c_m, leak g_l, or maximal channel conductance are not provided per unit membrane area, as in the morphological models, but simply as a pure capacitance or conductance associated with the compartment. For notational clarity we have omitted the compartment index from these parameters, but in NEAT they are specified on a per-compartment basis. Comparing this equation with the cable model (1), it can be seen that the 2nd-order spatial derivative is replaced by a sum of coupling terms $g_{ij}\,(v_i(t)-v_j(t))$, where g_{ij} is the coupling conductance between compartments i and j, and where $\mathcal{N}_i$ denotes the set of neighbour compartments to i. Compartmental models in NEAT can be derived in two ways: *(i)* through the 2nd-order finite difference approximation, capturing the full morphology in a detailed manner, and *(ii)* through Wybo's simplification algorithm, yielding reduced compartmental models.

S3.2 2nd-order finite difference approximation

Given an equispaced grid $\{x_i\}$ with spacing Δx, the finite difference approximation to the spatial 2nd-order derivative is given by:

$$\frac{\partial^2 v}{\partial x^2}(x,t) \approx \frac{v(x_{i+1},t) - 2v(x_i,t) + v(x_{i-1},t)}{\Delta x^2}. \tag{40}$$

Substituting this in (1) leads to a model of the form (39), where the relation between between cable parameters and compartment parameters has a straightforward interpretation (Pearlmutter and Zador 1998): membrane parameters – defined per unit area – are multiplied with a cylindrical surface, i.e $c_m \to 2\pi a \Delta x\, c_m, g_l \to 2\pi a \Delta x\, g_l, \overline{g}_c \to 2\pi a \Delta x\, \overline{g}_c$, and coupling conductances between compartments are proportional to cylindrical cross section and inversely proportional to inter-compartment distance, i.e. $g_{i,i-1} = g_{i,i+1} = \frac{\pi a^2}{r_a\,\Delta x}$ (Fig S1A).

At the junction of compartments, NEAT uses a discretization scheme that has empirically been observed to converge to the same parameter values as those yielded by the simplification toolchain (Fig S1B-D). Note that this scheme differs from the schemes implemented by NEURON and/or Arbor. To understand this discretization scheme, it is useful to note that the boundary condition (6), for a single child cylinder $\mathcal{C}_n = \{m\}$ and equal parameter values in nodes n and m, approximates the 2nd-order derivative

$$\begin{aligned} 0 &= -\frac{a_n^2}{r_{a,n}} \frac{\partial v_n}{\partial x}(x,t)\Big|_{x=L_n} + \frac{a_m^2}{r_{a,m}} \frac{\partial v_m}{\partial x}(x,t)\Big|_{x=0} \\ &\approx -\frac{a_n^2}{r_{a,n}} \frac{v_n(L_n,t) - v_n(L_n-\Delta x,t)}{\Delta x} \\ &\quad + \frac{a_m^2}{r_{a,m}} \frac{v_m(\Delta x,t) - v_m(0,t)}{\Delta x}. \end{aligned} \tag{41}$$

This can be seen from (5), which stipulates that $v_n(L_n,t) \equiv v_m(0,t)$. Note that this equation also models a compartment with zero total current (i.e. no capacitave, leak and channel currents). The geometrical interpretation can then be leveraged, assigning current to this compartment by taking half of the currents on the cylindrical surfaces between the adjacent compartments, yielding e.g. a leak that corresponds to $g_l = \pi a\, g_{ln} + \pi a\, g_{lm}$. For equal parameters in nodes n and m, this corresponds to the normal finite difference value $2\pi a g_{l,n}$. NEAT's discretization scheme

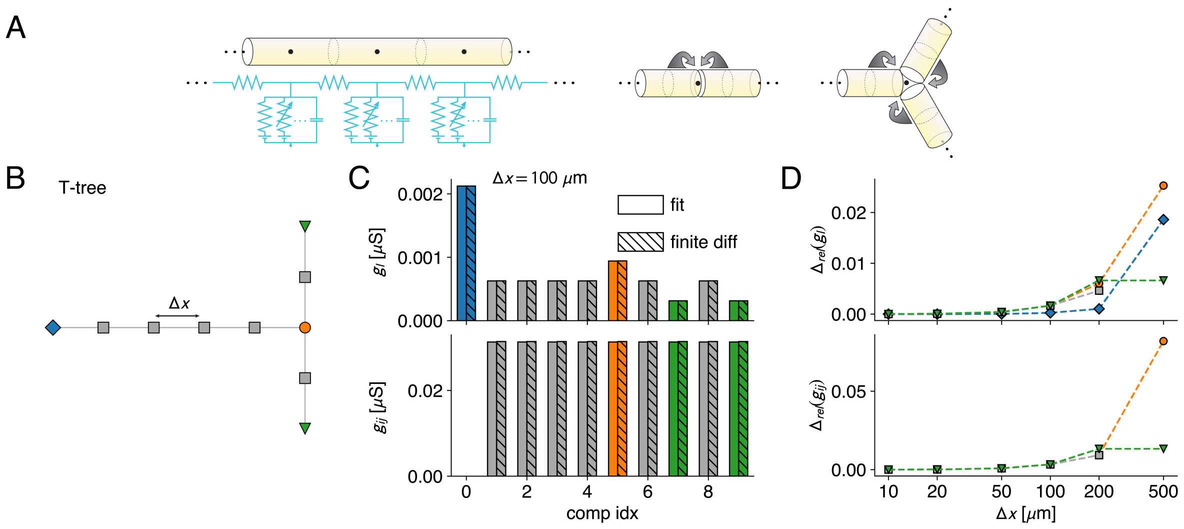

Fig. S1 NEAT's 2nd order finite difference approximation. **A:** In the geometrical interpretation of the 2nd order finite difference approximation, cylinders are subdivided into equally spaced sections, where the effective compartment locations in the are in the middle of each cylinder (left). Compartmental currents are computed as the product of current density and cylinder surface. An interpretation is that each compartment location is assigned half the current density on the cylindrical segments separating it from its neighbours (middle). This scheme is extended for bifurcation compartments (left). Coupling conductances are inversely proportional to the separation between compartments. **B:** Demonstration of the principles of the discretization on a simple T-morphology, featuring a soma (blue diamond) and a single dendrite that branches at a bifurcation (orange circle), terminating in two leaf compartments (green triangles). **C:** NEAT's discretization scheme converges between compartmental models derived through the reduction methodology (plain bars) and its 2nd order finite difference approximation (hatched bars). The leak conductance (top) at the bifurcation compartment (orange) is 3/2's the conductance of a normal compartent (grey), and 1/2 that of a normal compartment at the leafs, in agreement with the geometrical interpretation (A). Coupling conductances (bottom) in this scheme are equal for all compartments, as they are equally spaced, and again agree between reduction and finite difference approximation. **D:** Relative difference in parameter values between fit ($p_{\rm fit}$) and finite difference ($p_{\rm fd}$), i.e. $\Delta_{\rm rel}(p) = 2|p_{\rm fit} - p_{\rm fd}|/(p_{\rm fit} + p_{\rm fd})$, as a function of separation between compartments. Colors indicate compartment type (soma, on a linear dendrite, at bifurcation, or leaf compartment) as in B, C.

is therefore as follows: given a maximum inter-compartment distance $\Delta x_{\max}$, NEAT finds the maximal spacing $\Delta x \leqslant \Delta x_{\max}$, so that the minimum amount of compartments can be distributed on node n in an equispaced manner at locations $x = \Delta x, 2\Delta x, \ldots, L_n - \Delta x, L_n$. Coupling conductances between compartments are computed as $\frac{\pi a^2}{r_a \Delta x}$, and each compartment receives half the membrane currents that are on the cylindrical surfaces between the compartment and its neighbours (e.g. $g_l = \pi a_n \, g_{ln} + \sum_{m \in \mathcal{C}_n} \pi a_m \, g_{lm}$ for the leak conductance at a bifurcation).

S3.3 The simplification toolchain to obtain reduced compartmental models

NEAT implements Wybo's reduction methodology (Wybo et al. 2021), which is now extended to included concentration mechanism parameters. Given a set of compartment locations $\{\xi_i \,|\, i = 1, \ldots, N_c\}$ on the morphology, our fit process consists of the following steps: (1) fit the passive leak and coupling conductances, (2) fit the capacitances, (3) fit the maximal conductances of the ion channels, (4) fit the concentration mechanism parameters and (5) fit the leak reversal potentials to obtain the same resting membrane potentials as in the biophysical model.

1. To fit the passive leak and coupling conductances of the reduced model, NEAT first computes equilibrium voltage and concentrations throughout the detailed model, and creates a passified copy of the model. For this passified model, NEAT computes the matrix $Z^{N_c \times N_c}$, where $(Z)_{ij} = \tilde{Z}(\xi_i, \xi_j, \omega = 0)$. Note that this matrix relates steady-state input perturbation $\delta \mathbf{i} = (\delta i_1, \ldots, \delta i_{N_c})$ to voltage response

$\delta\mathbf{v} = (\delta v_1, \ldots, \delta v_{N_c})$, i.e. $\delta\mathbf{v} = Z\delta\mathbf{i}$. The fit then determines the admissible coupling terms between compartment sites from the original morphology, and creates a parametric matrix G of leak and coupling conductances. This matrix relates voltage response to input current, i.e. $G\delta\mathbf{v} = \delta\mathbf{i}$. Thus, for a perfect fit, G should be the inverse of the inverse of Z, and therefore $ZG \approx \mathbb{I}$, with $\mathbb{I}$ the identity matrix, is the fit objective, which NEAT solves in the linear least-squares sense.

2. NEAT fits the capacitances by matching the largest response time-scale of the full model, obtained from (29), to the largest response time-scale of the reduced model.
3. NEAT fits the maximal conductances of each ion channel type separately. To that purpose, NEAT creates copies of the original model where all but one of the channels are removed. For each of these channels c, and for a number (K) of voltage and state variable expansion points, NEAT then computes the $Z_{c1}, \ldots, Z_{cK}$ under the quasi-active approximation. If the state variable depends on the Ca^{2+}-concentration, NEAT substitutes the equilibrium concentration. NEAT then uses quasi-active approximations in the compartmental model, around the same expansion points as in the full model, to obtain K conductance matrices. These matrices can be decomposed as the sum of G_{pas}, a matrix containing the passive conductances fitted in step 1, and a diagonal matrix $G_{c,k}$, containing on its diagonal the quasi active terms associated with channel c, which in turn feature the to be fitted maximal conductance parameters for each compartment. The fit objective is given by $(G_{\text{pas}} + G_{c,k})\, Z_{c,k} \approx \mathbb{I}$, and is again solved in the linear least-squares sense.
4. Concentration mechanisms, specifically Ca^{2+}, are typically described by an equation of the form $\frac{d[Ca^{2+}]}{dt} = -\frac{[Ca^{2+}]}{\tau} + \gamma i_{\text{Ca}}$, with $[Ca^{2+}]$ the Ca^{2+}-concentration, τ the decay time constant of this concentration, γ a fit parameter influencing how strongly Ca^{2+}-channels change the Ca^{2+}-concentration, and i_{Ca} the total current through all Ca^{2+}-channels. NEAT fits the γ_{red}-parameter of the Ca^{2+} in the reduced model to achieve the same concentration in the reduced compartment as at the corresponding site in the mophological model. To that purpose, NEAT sets $\gamma_{\text{red}} = \gamma_{\text{orig}} \frac{\overline{g}_{\text{Ca,orig}}}{\overline{g}_{\text{Ca,red}}}$, with $\overline{g}_{\text{Ca,orig}}$ the total maximal conductance densitgy of all Ca^{2+}-channels at the compartment site in the full model, and $\overline{g}_{\text{Ca,red}}$ the total maximal conductance of the Ca^{2+}-channels at the reduced compartment, which was determined in step 3.
5. Finally, NEAT fits the leak reversals e_{li} for each compartment $i = 1, \ldots, N_c$ to reproduce the equilibrium voltage $v_{eq}(\xi_i)$ at the corresponding compartment site in the full model. To achieve this, NEAT evaluates all voltages, concentrations and channel state variables in the full model at equilibrium at all compartment sites, substitutes them in (39) while setting input current i_i and temporal derivative $\dot{v}_i$ to zero. In this way a system with N_c equations is obtained that is linear in the N_c uknowns e_{li}. This system is then solved using standard Gauss-Jordan elimination.

S4 Simulating the neuron models

Neither NEURON nor Arbor provide direct access to set compartmental parameters; while all simulations of morphological neurons are based on the compartmental description (39), the precise spatial discretization scheme is implemented by the simulator itself. For instance, the NEURON book (Carnevale and Hines 2004) suggests that maximal compartment spacing should be determined as a user-defined fraction of the frequency dependent length constant $\lambda(\omega)$, evaluated at 100 Hz. In these simulators, one can either read in morphologies, which are then internally mapped to compartments, or construct neuron models as concatenations of cylindrical sections, which are then discretized internally. Model exports to simulators in NEAT are built to maximize internal consistency with the various analytical frameworks. To that purpose, NEAT proposes two ways of exporting models: either as systems of coupled cylinders – which are most consistent with assumptions in the morphological neuron models, and are then discretized internally by the simulator – or directly as compartmental models – for which the exact compartmental parameters are defined by NEAT.

S4.1 Exporting morphological models to NEURON

To export morphological models to NEURON, NEAT implements the `NeuronSimTree` class as a subclass of `PhysTree`. Based on the geometrical and physiological parameters stored at each node, a cylindrical `Section` is created in NEURON, that is then connected to its parent section. This cylindrical section is by default discretized following NEURON's heuristic based on the frequency-dependent length constant $\lambda(\omega = 100 \text{ Hz})$.

S4.2 Exporting compartmental models to NEST and NEURON

NEAT implements functionality to export compartmental models to both NEST and NEURON. NEST was recently extended with a compartmental modelling framework (Espinoza Valverde et al. 2024), where custom ion channels and synaptic receptors can be defined through the NESTML model description language (Linssen et al. 2023, 2024). As opposed to NEURON, this compartmental modelling framework exposes the parameters of single compartments. Therefore, the `NestCompartmentTree` is a straightforward extension of the `CompartmentTree`, which instantiates the compartmental model in NEST. The NEURON implementation, however, is somewhat more involved. It requires the creation of a cylindrical section for each compartment, whose surface area, length, membrane parameters, and axial resistance are chosen such that, when this cylindrical section is forced to contain only a single compartment, the compartmental parameters computed internally in NEURON will correspond with those of NEAT's `CompartmentTree`. These operations are implemented in the `NeuronCompartmentTree`. Note that NEAT has two ways of constructing compartmental models: through the simplification procedure implemented by the `fit_model()` function of `CompartmentFitter`, or through NEAT's 2nd order finite difference scheme, implemented by the `create_finite_difference_tree()` function of `PhysTree`. therefore, even though NEST's compartmental modelling framework has a low-level user interface, it can still be used to simulate highly detailed morphological models.

References

Butz, E.G., Cowan, J.D.: Transient potentials in dendritic systems of arbitrary geometry. Biophysical journal **14**, 661–689 (1974) https://doi.org/10.1016/S0006-3495(74)85943-6

Cao, B.J., Abbott, L.F.: A new computational method for cable theory problems. Biophysical Journal **64**(2), 303–313 (1993) https://doi.org/10.1016/S0006-3495(93)81370-5

Combe, C.L., Canavier, C.C., Gasparini, S.: Intrinsic Mechanisms of Frequency Selectivity in the Proximal Dendrites of CA1 Pyramidal Neurons. The Journal of Neuroscience **38**(38), 8110–8127 (2018) https://doi.org/10.1523/JNEUROSCI.0449-18.2018

Carnevale, N.T., Hines, M.L.: The NEURON Book. Cambridge University Press (2004) https://doi.org/https://doi.org/10.1017/CBO9780511541612

Coombes, A.S., Timofeeva, Y., Svensson, C., Lord, G.J., Josić, K., Cox, S.J., Colbert, C.M., Josi, K.: Branching dendrites with resonant membrane : A "sum-over-trips" approach. Biological Cybernetics **97**(2), 137–149 (2007) https://doi.org/10.1007/s00422-007-0161-5

Das, A., Rathour, R.K., Narayanan, R.: Strings on a Violin: Location Dependence of Frequency Tuning in Active Dendrites. Frontiers in Cellular Neuroscience **11** (2017) https://doi.org/10.3389/fncel.2017.00072

Espinoza Valverde, J.A., Müller, E., Haug, N., Schöfmann, C.M., Linssen, C., Senk, J., Spreizer, S., Trensch, T., Lober, M., Jiang, H., Kurth, A., Acimovic, J., Korcsak-Gorzo, A., Welle Skaar, J.-E., Terhorst, D., Stapmanns, J., Graber, S., Schepper, R., Eppler, J.M., Kunkel, S., Mitchell, J., Wybo, W., Morrison, A., Vogelsang, J., Benelhedi, M.A., Köhn, C., Schmitt, F., Manninen, T., Albada, S., Gille, J., Jenke, J., Plesser, H.E.: NEST 3.7. https://doi.org/10.5281/zenodo.10834751 . https://doi.org/10.5281/zenodo.10834751

Gustavsen, B., Semlyen, A.: Simulation of transmission line transients using vector fitting and modal decomposition. IEEE Transactions Power Delivery **13**(2), 605–614 (1998) https://doi.org/10.1109/61.660941

Gustavsen, B., Semlyen, A.: Rational approximation of frequency domain responses by vector fitting. IEEE Transaction on Power Delivery **14**(3), 1052–1061 (1999) https://doi.org/10.1109/61.772353

Hodgkin, A.L., Huxley, A.F.: A quantitative description of membrane current and its application to conduction and excitation in nerve. The Journal of physiology **117**(4), 500–544 (1952) https://doi.org/10.1113/jphysiol.1952.sp004764

Holmes, W.R., Segev, I., Rall, W.: Interpretation of time constant and electrotonic length estimates in multicylinder or branched neuronal structures. Journal of neurophysiology **68**(4), 1401–20 (1992) https://doi.org/10.1152/jn.1992.68.4.1401

Koch, C.: Biophysics of Computation: Information Processing in Single Neurons. Oxford University Press (1998). https://doi.org/10.1093/oso/9780195104912.001.0001

Koch, C., Poggio, T.: A simple algorithm for solving the cable equation in dendritic trees of arbitrary geometry. Journal of neuroscience methods **12**(4), 303–315 (1985) https://doi.org/10.1016/0165-0270(85)90015-9

Kravanja, P., Van Barel, M.: Computing the Zeros of Analytic Functions. Lecture Notes in Mathematics. Springer (2000)

Linssen, C., Babu, P.N., Bouhadjar, Y., Ewert, L., Wybo, W., Lober, M., Feller, F., Rumpe, B., Morrison, A.: NESTML 8.0.0. https://doi.org/10.5281/zenodo.12191059 . https://doi.org/10.5281/zenodo.12191059

Linssen, C.A.P., Babu, P.N., Eppler, J.M., Ewert, L., Blaszczyk, K., Wybo, W., Strohmer, B., Rumpe, B., Morrison, A.: NESTML 7.0.0. https://doi.org/10.5281/zenodo.10125416 . https://doi.org/10.5281/zenodo.10125416

Laudanski, J., Torben-Nielsen, B., Segev, I., Shamma, S.: Spatially distributed dendritic resonance selectively filters synaptic input. PLoS computational biology **10**(8), 1003775 (2014) https://doi.org/10.1371/journal.pcbi.1003775

Major, G.: Solutions for transients in arbitrarily branching cables: III. Voltage clamp problems. Biophysical journal **65**(1), 469–491 (1993) https://doi.org/10.1016/S0006-3495(94)80836-7

Mauro, A., Conti, F., Dodge, F., Schor, R.: Subthreshold behavior and phenomenological impedance of the squid giant axon. The Journal of general physiology **55**(4), 497–523 (1970) https://doi.org/10.1085/jgp.55.4.497

Major, G., Evans, J.D.: Solutions for transients in arbitrarily branching cables: IV. Nonuniform electrical parameters. Biophysical journal **66**(3 Pt 1), 615–33 (1994) https://doi.org/10.1016/S0006-3495(94)80836-7

Major, G., Evans, J.D., Jack, J.B.: Solutions for transients in arbitrarily branching cables: I. Voltage recording with a somatic shunt. Biophysical journal **65**(1), 423–49 (1993) https://doi.org/10.1016/S0006-3495(93)81037-3

Major, G., Evans, J.D., Jack, J.B.: Solutions for transients in arbitrarily branching cables II. Voltage clamp theory. Biophysical journal **65**(1), 469–491 (1993) https://doi.org/10.1016/S0006-3495(94)80836-7

Monai, H., Omori, T., Okada, M., Inoue, M., Miyakawa, H., Aonishi, T.: An analytic solution of the cable equation predicts frequency preference of a passive shunt-end cylindrical cable in response to extracellular oscillating electric fields. Biophysical journal **98**(4), 524–33 (2010) https://doi.org/10.1016/j.bpj.2009.10.041

Pearlmutter, B.A., Zador, A.: In: Koch, C. (ed.) Biophysics of Computation: Information Processing in Single Neurons (Computational Neuroscience). Oxford University Press (1998). https://doi.org/10.1093/oso/9780195104912.001.0001

Rall, W.: Branching dendritic trees and motoneuron membrane resistivity. Experimental Neurology **1**(5), 491–527 (1959) https://doi.org/10.1016/0014-4886(59)90046-9

Rall, W.: Membrane potential transients and membrane time constant of motoneurons. Experimental Neurology **2**(5), 503–532 (1960) https://doi.org/10.1016/0014-4886(60)90029-7

Rall, W.: Electrophysiology of a Dendritic Neuron Model. Biophysical Journal **2**(2), 145–167 (1962) https://doi.org/10.1016/S0006-3495(62)86953-7

Stakgold, I.: Boundary Value Problems of Mathematical Physics. Macmillan series in advanced mathematics and theoretical physics, vol. v. 1. Macmillan (1967) https://doi.org/https://doi.org/10.1137/1.9780898719888

Timofeeva, Y., Coombes, S.: In: Cuntz, H., Remme, M.W.H., Torben-Nielsen, B. (eds.) Response of Gap Junction-Coupled Dendrites: A Sum-Over-Trips Approach, pp. 449–464. Springer, New York, NY (2014) https://doi.org/10.1007/978-1-4614-8094-5_27.

Tuckwell, H.C.: Introduction to Theoretical Neurobiology. Cambridge Studies in Mathematical Biology. Cambridge University Press (1988). https://doi.org/https://doi.org/10.1017/CBO9780511623271

Ulrich, D.: Dendritic resonance in rat neocortical pyramidal cells. Journal of neurophysiology **87**(6), 2753–9 (2002) https://doi.org/10.1152/jn.01000.2001

Vaidya, S.P., Johnston, D.: Temporal synchrony and gamma-to-theta power conversion in the dendrites of CA1 pyramidal neurons. Nature neuroscience **16**(12), 1812–1820 (2013) https://doi.org/10.1038/nn.3562 24185428

Wybo, W.A., Jordan, J., Ellenberger, B., Marti Mengual, U., Nevian, T., Senn, W.: Data-driven reduction of dendritic morphologies with preserved dendro-somatic responses. eLife **10**, 60936 (2021) https://doi.org/10.7554/eLife.60936

Wybo, W.A.M., Torben-Nielsen, B., Nevian, T., Gewaltig, M.O.: Electrical Compartmentalization in Neurons. Cell Reports **26**(7), 1759–17737 (2019)

https://doi.org/10.1016/j.celrep.2019.01.074

Watanabe, H., Tsubokawa, H., Tsukada, M., Aihara, T.: Frequency-dependent signal processing in apical dendrites of hippocampal CA1 pyramidal cells. Neuroscience **278**, 194–210 (2014) https://doi.org/10.1016/j.neuroscience.2014.07.069
